# Phylogenomic disentangling of the *Bifidobacterium longum* subsp. *infantis* taxon

**DOI:** 10.1099/mgen.0.000609

**Published:** 2021-07-28

**Authors:** Chiara Tarracchini, Christian Milani, Gabriele Andrea Lugli, Leonardo Mancabelli, Federico Fontana, Giulia Alessandri, Giulia Longhi, Rosaria Anzalone, Alice Viappiani, Francesca Turroni, Douwe van Sinderen, Marco Ventura

**Affiliations:** ^1^​ Laboratory of Probiogenomics, Department of Chemistry, Life Sciences, and Environmental Sustainability, University of Parma, Parma, Italy; ^2^​ Microbiome Research Hub, University of Parma, Parma, Italy; ^3^​ GenProbio Srl, Parma, Italy; ^4^​ APC Microbiome Ireland and School of Microbiology, Bioscience Institute, National University of Ireland, Cork, Ireland

**Keywords:** bifidobacteria, bacterial evolution, comparative genomics, infant gut microbiome

## Abstract

Members of the *Bifidobacterium longum* species have been shown to possess adaptive abilities to allow colonization of different mammalian hosts, including humans, primates and domesticated mammalian species, such as dogs, horses, cattle and pigs. To date, three subspecies have formally been recognized to belong to this bifidobacterial taxon, i.e. *B. longum* subsp. *longum*, *B. longum* subsp. *infantis* and *B. longum* subsp. *suis*. Although *B. longum* subsp. *longum* is widely distributed in the human gut irrespective of host age, *B. longum* subsp. *infantis* appears to play a significant role as a prominent member of the gut microbiota of breast-fed infants. Nevertheless, despite the considerable scientific relevance of these taxa and the vast body of genomic data now available, an accurate dissection of the genetic features that comprehensively characterize the *B. longum* species and its subspecies is still missing. In the current study, we employed 261 publicly available *B. longum* genome sequences, combined with those of 11 new isolates, to investigate genomic diversity of this taxon through comparative genomic and phylogenomic approaches. These analyses allowed us to highlight a remarkable intra-species genetic and physiological diversity. Notably, characterization of the genome content of members of *B. longum* subsp. *infantis* subspecies suggested that this taxon may have acquired genetic features for increased competitiveness in the gut environment of suckling hosts. Furthermore, specific *B. longum* subsp. *infantis* genomic features appear to be responsible for enhanced horizontal gene transfer (HGT) occurrences, underpinning an intriguing dedication toward acquisition of foreign DNA by HGT events.

## Data Summary

Decoded genome sequences of 11 newly isolated *B. longum* strains were deposited at NCBI database under BioProject code PRJNA692178. A full listing of NCBI accession data for *B. longum* strains described in this paper is available in Table S1 (available in the online version of this article).

Supplementary material can be found at 10.6084/m9.figshare.14448249.

**Impact Statement**

In this study, through comparative genomic analyses and phylogenomic reconstruction of 261 publicly available *B. longum* genomes, we gained insight into intra-species genetic and physiological diversity, identifying specific *B. longum* subsp. *infantis* genomic features, which appear to be linked with its enhanced ability to acquire foreign DNA. This remarkable genome plasticity may contribute to explain the specific adaptation of *B. longum* subsp. *infantis* toward colonization of the gut of suckling mammals.

## Introduction

The human gut harbours at least 100 trillion (10^14^) microbial cells [1], collectively organized in a complex and dynamic microbial community that plays a fundamental role in defining the human health status [2]. It is well known that members of the gut microbiota engage in complex microbe–microbe and microbe–host interactions, with physiological consequences, including participation in metabolic activities such as (sometimes syntrophic) degradation of non-digestible carbohydrates, with consequent production of short-chain fatty acids (SCFAs) [3, 4]. The assembly of the human gut microbiota is believed to commence during delivery when the newborn passes through the mother’s birth canal [5]. During the developmental period following birth, the early gut microbiota is influenced by various factors, including mode of delivery, duration of gestation, antibiotic exposure, as well as feeding type [6, 7]. This latter factor is particularly noteworthy since breast-feeding can shape the gut-microbiota composition of the newborn by promoting a microbial community enriched by members of the *Bifidobacterium* genus [8]. In addition to the fermentation of non-digestible food compounds, especially glycans, the (bifido)bacterial consortia also engage with the host immune system, stimulating and modulating both innate and adaptive host immune responses, ultimately influencing overall intestinal functionality and homeostasis [9, 10]. Interestingly, it has been reported that particular bifidobacterial species, such as *Bifidobacterium longum* subsp. *infantis*, *Bifidobacterium bifidum* and *Bifidobacterium breve*, are able to efficiently utilize (certain) human milk oligosaccharides (HMOs) [11–15]. HMOs constitute complex milk glycans known to elicit prebiotic activity by allowing the above-mentioned bifidobacterial species to establish and persist in the infant gut, thereby representing a clear example of host-microbe co-evolution in humans [16–20].

Members of the *Bifidobacterium longum* species have been identified as very common inhabitants of the mammalian gut, reaching a prevalence of 95.5 %, representing the percentage of individuals harbouring this species within the population, as shown by a recent survey conducted in 67 assessed mammalian hosts [21]. In recent decades, members of the *B. longum* species have been grouped into three distinct subspecies**,** i.e. *Bifidobacterium longum* subsp. *longum*, *Bifidobacterium longum* subsp. *infantis* and *Bifidobacterium longum* subsp. *suis* [22], the latter isolated from the gut microbiota of swine [22, 23]. Despite the progressive reduction in the relative abundance of bifidobacteria in the human gut starting from 1/2 years of age [7], members of *B. longum* subsp. *longum* are known to commonly inhabit the infant, adult and elderly human gut [24], thereby perhaps exerting their positive health footprint throughout the human lifespan [24, 25]. In contrast, *B. longum* subsp. *infantis* is most frequently isolated from breast-fed infant faeces [26, 27]. Consistently, the decoding of *B. longum* subsp. *infantis* ATCC15697 genome sequence, which was published in 2008, revealed a genome that is dedicated to the degradation and utilization of a wide range of HMOs [15, 28].

Due to the substantial scientific and commercial interest in members of this species, which are able to colonize different hosts at different stages of life, during which they may contribute to host health, a large number of *B. longum* strains have been sequenced. Nevertheless, a comprehensive dissection of the genetic potential of *B. longum* and its subspecies is still lacking. For this reason, we decided to investigate the genomic diversity of and phylogenetic relationships between members of the *B. longum* species. This prompted a complete revision of subspecies classification and allowed a detailed dissection of their genetic features presumed to be responsible for efficient niche adaptation.

## Methods

### Ethical statement

Animal research was performed in compliance with the rules, regulations and recommendations of the Ethical Committee of the University of Parma. The corresponding protocols were approved by the ‘Comitato di Etica Università degli Studi di Parma’, Italy. All animal procedures were carried out in accordance with national guidelines (Decreto legislativo 26/2014).

Furthermore, the human study protocol (protocol number 2016/0028558) was approved by the Ethics Committee of the ‘Azienda Unità Sanitaria Locale di Reggio Emilia ‐ IRCCS’ in Reggio Emilia, Italy, as well as by the Ethics Committee of the University of Parma, Italy, and informed written consent was obtained from all participants or their legal guardians.

### *B. longum* genome sequences

At the time of writing (November 2020), 363 publicly available *B. longum* genomes (complete and draft genome sequences) were retrieved from the National Center for Biotechnology Information (NCBI) public database and then subjected to genome quality-based selection. In detail, genome sequences showing a genome size less than 2.20 Mb or/and with a number of predicted CDSs less than 1600 as well as those exhibiting low sequencing quality (genome coverage lower than 30-fold or containing more than 100 contigs) were manually identified and discarded. Furthermore, duplicated bacterial genomes (ANI value >99.99 %) were removed, resulting in a final collection of 261 high-quality *B. longum* genomes encompassing 243, 7 and 11 chromosomes belonging to *B. longum* subsp. *longum*, *B. longum* subsp. *suis* and *B. longum* subsp. *infantis* subspecies, respectively. Furthermore, we decoded the chromosomes of 11 newly isolated *B. longum* strains that were also included in this study (Table S1). Notably, these latter isolates were obtained from human, bovine and canine faecal samples within the context of a bifidobacterial strain isolation project aimed at exploring the genetic variability of the *Bifidobacterium* genus.

### Identification of novel *B. longum* strains and chromosomal DNA extraction

Based on a previous cultivation effort aimed at isolating *Bifidobacterium pseudolongum* strains from faecal samples of various mammalian species [29], bifidobacterial strains that did not belong to the above-mentioned bifidobacterial species were further subjected to species-specific PCR-based characterization in order to identify novel *B. longum* strains. Briefly, bifidobacterial strains were incubated in an anaerobic atmosphere (2.99 % H_2_, 17.01 % CO_2_ and 80 % N_2_) in a chamber (Concept 400, Ruskinn) in de Man-Rogosa-Sharpe (MRS) (Sharlau Chemie) supplemented with 0.05 % (wt/vol) l-cysteine hydrochloride and incubated at 37 °C for 16 h. Subsequently, cells were harvested by centrifugation at 3500 ***g*** for 8 min, and the obtained cell pellet was used for DNA extraction using the GenEluteTM Bacterial Genomic DNA kit (Merck, Germany), following the manufacturer’s instructions. The extracted DNA was then subjected to a *B. longum* species-specific identification protocol through a PCR-based methodology using primers Blong1 5′-TCCCAGTTGATCGCATGGTC-3′ and Blong2 5′-GGGAAGCCGTATCTCTACGA-3′, which are based on the 16S rRNA gene sequences of this taxon [30]. PCR amplification was carried out according to the following protocol: one cycle of 94 °C for 5 min, followed by 30 cycles of 94 °C for 30 s, 54 °C for 30 s and 72 °C for 50 s, and a final cycle of 72 °C for 5 min. Furthermore, the DNA of strains identified as *B. longum* ssp. were further subjected to a genotyping PCR using primers ERIC1 5′-ATGTAAGCTCCTGGGGATTCAC-3′ and ERIC2 5′-AAGTAAGTGACTGGGGTGAGCG-3′ in order to sequence the genome of only one representative per genotype [31]. PCR amplification was performed according to a previous protocol: one cycle at 94 °C for 3 min, followed by 35 cycles of 94 °C for 30 s, 48 °C for 30 s and 72 °C for 4 min, and a final cycle at 72 °C for 10 min [31].

### *B. longum* genome sequencing and assemblies

Chromosomal DNA of the 11 newly identified *B. longum* strains was sequenced by GenProbio Srl (http://genprobio.com) using a MiSeq platform (Illumina, San Diego, CA, USA) according to the supplier’s protocol employing the Nextera XT DNA Library Prep Kit (Illumina), resulting in fragments of about 500–900 bp. The library samples obtained were then pooled into a Flow Cell V3 600 cycle (Illumina) in order to retrieve paired-end reads of 250 bp resulting from sequencing of fragment ends. Fastq files of paired-end reads generated from each genome sequencing effort were used as input for the genome assembly through the MEGAnnotator pipeline (https://github.com/GabrieleAndrea/MEGAnnotator) [32]. The SPAdes v3.14.0 program included in the MEGAnnotator platform was used for *de novo* assembly of each bifidobacterial genome sequence with the pipeline option ‘--careful’ and a list of k-mer sizes 21,33,55,77,99,127 as suggested in the SPAdes’ manual [33]. MEGAnnotator then employed contigs greater than 1000 bp to predict protein-encoding ORFs using Prodigal v2.0 (Linux command line ‘./prodigal -f gff -a [protein_translation_to_selected_file] -i [input_filename.fasta] -o [output_filename]”) [34]. Predicted ORFs were then functionally annotated using RAPSearch2 (reduced alphabet-based protein similarity search) (cutoff *e*-value of 1×10^−5^ and minimum alignment length 20) employing the NCBI reference sequences (RefSeq) database [35] together with hidden Markov model profile (HMM) searches (http://hmmer.org/) performed against the manually curated Pfam-A database (cutoff *e*-value of 1×10^−10^).

### Pan-genome analyses of *B. longum* genomes

All 272 genome sequences of *B. longum* were employed for a core-genome analysis using the Pangenome Analysis Pipeline (PGAP) v1.1 (Linux command line ‘./PGAP.pl --strains [input_strain_list] --input input_path/ --output output_path/ --thread 20 --identity 0.5 --coverage 0.8 --cluster --method GF’) (http://pgap.sf.net) [36]. Predicted CDSs of each *B. longum* genome were classified into functional gene clusters through the gene family (GF) method, consisting of pairwise protein-similarity search employing blast software v2.2.28+ (cutoff *e*-value of 1×10^−10^ and exhibiting at least 50 % identity across at least 80 % of both protein sequences). Following this, using MCL (graph-theory-based Markov clustering algorithm) [37], the data obtained were used to assign proteins to so-called Clusters of Orthologous Groups (COGs). A pan-genome profile was then built using an optimized algorithm as part of the pgap software v1.1, based on a presence/absence matrix encompassing all COGs identified in the analysed genomes (Linux command line ‘./PGAP.pl --strains [input_strain_list] --input input_path/ --output output_path/ --thread 20 --identity 0.5 --coverage 0.8 --cluster --method GF --evolution --pangenome’). Subsequently, the core genome of *B. longum* species was obtained by selecting protein families, which are shared between all genomes, while truly unique genes (TUGs) encoded by a single genome were identified based on those protein families that are present in one *B. longum* genome yet absent in all other *B. longum* genomes. Separate pan- and core-genome analyses were performed on each *B. longum* subspecies as described above, involving genomes of 251 *B. longum* subsp. *longum*, 11 *B. longum* subsp. *infantis* and ten *B. longum* subsp. *suis* genomes.

### Phylogenomic comparison between *B. longum* strains

**Fig. 1.**
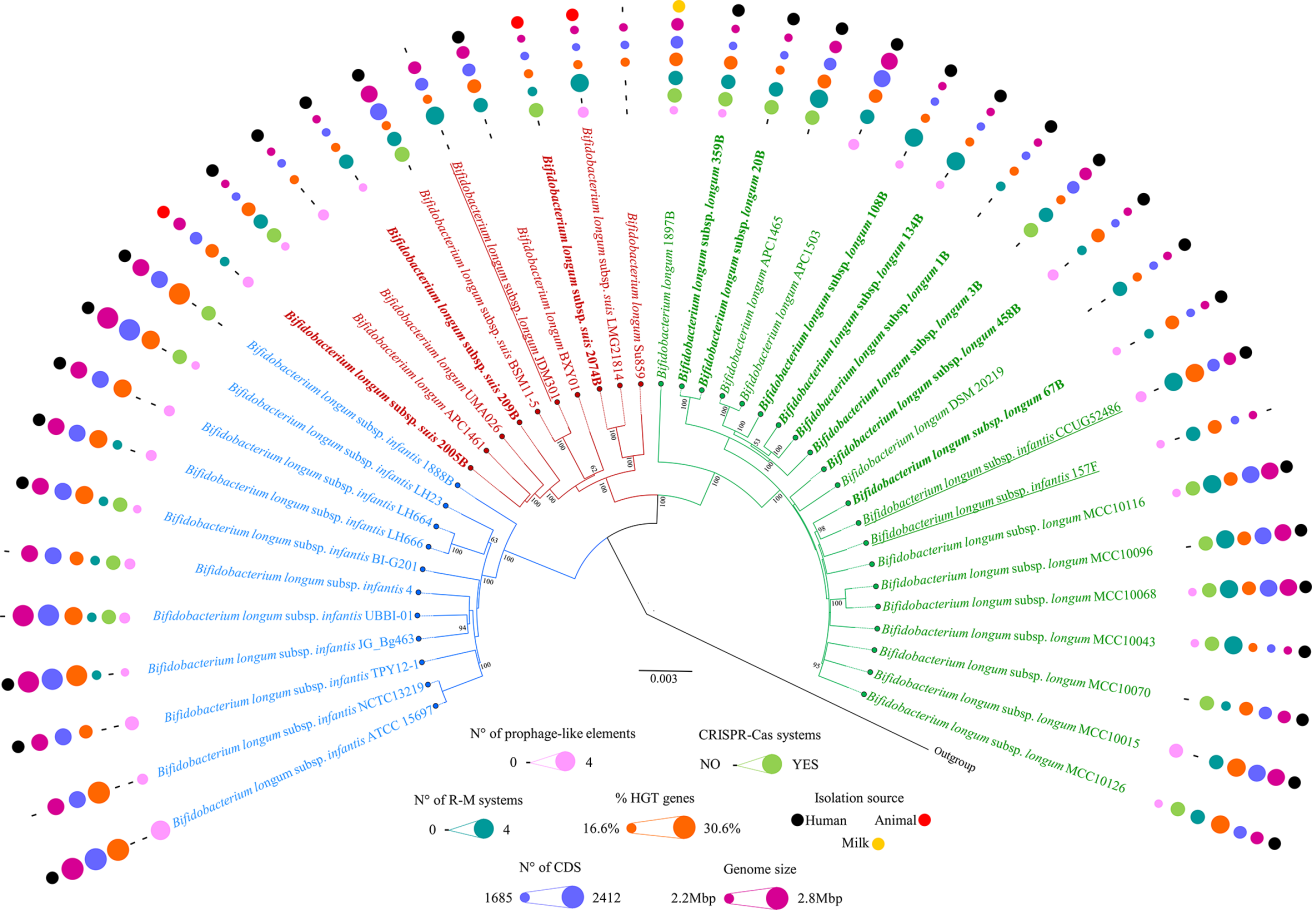
Phylogenomic tree based on the core genome of *B. longum* species. The phylogenomic tree, showing a selection of 42 representative genomes belonging to the *B. longum* species, was based on the concatenation of the 510 *B. longum* core genes and was built through the neighbour-joining method. Bootstrap percentages above 50 are shown at node points, based on 1000 replicates. Misclassified strains were underlined while the 11 new isolates were highlighted in bold. Phylogenetic clusters are highlighted with similarly coloured branches. Circles surrounding the tree represent the genome sizes (in dark pink), numbers of CDS (in purple), percentages of genes predicted to have undergone horizontal gene transfer (in orange), number of R-M systems (in dark green), occurrence of CRISPR-Cas systems (in light green), and isolation source (black=human, red=animal, yellow=milk).

In order to assess the genetic relatedness among the 272 members of *B. longum* species, the COGs constituting the core genome of each *B. longum* strain were concatenated, and they were then aligned using mafft v7.222 [38] through the Linux command line ‘mafft --thread 20 --retree 2 --clustalout --reorder [input_sequences.fasta] >output.aln’. The resulting phylogenomic tree was constructed using the neighbour-joining method in ClustalW v2.1 [39] through the Linux command line ‘clustalw -bootstrap=100 -seed=100 -bootlabels=NODE -outputtree=phylip -infile=file.aln’. Then, utilizing the graphical viewer of phylogenetic trees FigTree v1.4 (http://tree.bio.ed.ac.uk/software/figtree/), the core-genome-based visual tree was developed. Furthermore, a value for the average nucleotide identity (ANI) was calculated for each genome pair using the fastANI software v1.3 [40] through the Linux command line ‘./fastANI --ql [genome_list_path] --rl [genome_list_path] -t 20 --matrix -o output.txt’. Out of 272 obtained *B. longum* genomes, we selected 42 *B. longum* strains in order to perform downstream analyses (Fig. 1). For this purpose, we included all ten genomes that clustered with the *B. longum* subsp. *suis* type strain DSM20097 (seven publicly available and three newly isolated), 11 of the non-redundant identified *B. longum* subsp. *infantis* chromosomes with suitable quality (see above), along with 21 representative of *B. longum* subsp. *longum*. Notably, these latter comprised the type strain DSM 20219, eight newly isolated, and an additional 12 publicly available genome sequences, selected to maximize the description of the intra-subspecies diversity from the branch of the tree encompassing the whole selection of *B. longum* subsp. *longum* (Fig. S2).

### Prediction of the mobilome of *B. longum*

The identification of the genes that may have been acquired by horizontal gene transfer (HGT) events was achieved using the suite colombo v3.8, with a sensitivity value of 0.7 (https://github.com/brinkmanlab/colombo/releases) [41]. Furthermore, the proteome of each *B. longum* strain was screened for the presence of restriction-modification (R-M) systems based on sequence similarity to genes classified in the rebase database [42] (http://rebase.neb.com/rebase/rebhelp.html; blast cutoff *e*-value of 1×10^−5^). The presence of transposable elements was performed through the IS Finder online tool with predefined parameters (https://isfinder.biotoul.fr/), while identification of clustered regularly interspaced short palindromic repeats (CRISPRs) was achieved through the web application CRISPRfinder (https://crispr.i2bc.paris-saclay.fr/Server/; default parameters were used) [43]. Prediction of prophage-like elements was conducted using a custom blast database (cutoff *e*-value of 1×10^−5^) encompassing previously bifidoprophage-validated sequences obtained from bifidobacterial type strains previously described [44]. Then, genomic regions encompassing predicted phage-related genes were manually examined to identify complete prophage-like sequences. Assessment of complete or partial plasmid sequences was carried out employing a combination of the PlasmidFinder 2.1 web service (https://cge.cbs.dtu.dk/services/PlasmidFinder/; minimum identity=50 % and minimum coverage=80 %) [45] and ABRicate software (https://github.com/tseemann/abricate).

### *B. longum* type strains carbohydrate growth assays

In order to validate the *in silico* findings, we performed growth assays on selected carbon sources involving the type strains of each *B. longum* subspecies, i.e. *B. longum* subsp. *longum* DSM20219, *B. longum* subsp. *suis* DSM20097 and *B. longum* subsp. *infantis* ATCC15697. Notably, *in silico* analyses performed in this study generated predictions with regards to (carbohydrate) metabolic abilities of the above-mentioned strains and further discussed in Results. *B. longum* type strains were cultivated overnight on semisynthetic MRS medium supplemented with 0.05 % (w/vol) l-cysteine hydrochloride at 37 °C under anaerobic conditions. Subsequently, cells were diluted in MRS without glucose in order to obtain an OD_600 nm_=1 and 15 µl of the diluted cells were inoculated in 135 µl of MRS without glucose supplemented with 1 % (wt/vol) of a particular sugar in a 96-well microtitre plate and incubated in an anaerobic cabinet. Specifically, each carbohydrate was dissolved in MRS without glucose previously sterilized by autoclaving at 121 °C for 15 min. Subsequently, each obtained solution was filter sterilized using a 0.2 µm filter size prior to use. Cell growth was evaluated by monitoring the optical density at 600 nm with the use of a plate reader (Biotek, VT, USA). The plate was read in discontinuous mode, with absorbance readings performed at 3 min intervals for three times after 48 h of growth, and each reading was ahead of 30 s of shaking at medium speed. Cultures were grown in triplicates, and the resulting growth data were expressed as the average of these replicates. Carbohydrates tested in this study were purchased from Merck (Germany) and Carbosynth (Berkshire, UK), and include soluble starch from potato, amylopectin from maize, pullulan, maltotriose, maltodextrin, FOS, d-(+)-maltose, d-(+)-xylose, 2′-Fucosyllactose (2′-FL), 3′-Sialyllactose (3′-SL), and α-d-glucose.

### Statistical analyses

All statistical analyses were performed with SPSS software v25 (www.ibm.com/software/it/analytics/spss/).

## Results and Discussion

### General genome features of *B. longum* genomes included in the comparative genomics analysis

In order to investigate the phylogenomic diversity of members belonging to the *B. longum* species*,* we undertook a comparative genomics analysis involving high-quality *B. longum* genome sequences selected amongst those publicly available (complete and draft genome sequences, see M and M section for the inclusion/exclusion criteria used). Remarkably, among the latter, *B. longum* subsp. *infantis* strains exhibited the highest number of suspected duplicated genomes (ANI ≥99.99 %). Accordingly, we removed such apparent copies of identical chromosomes, which had been deposited under different strain IDs, thereby allowing the generation of a curated *B. longum* subsp. *infantis* genome collection without duplicated chromosomal sequences (Table S1). The final collection of 272 *B. longum* genomes, including the 11 sequenced in this study, encompassed chromosomal sequences ranging in size from 2.2 Mb for *B. longum* APC1478 to 2.8 Mb for *B. longum* subsp. *infantis* ATCC 15697. As outlined in Table S1, the number of predicted coding DNA sequences (CDS) ranged from 1685 for *B. longum* subsp. *longum* 296B to 2412 for *B. longum* subsp. *infantis* ATCC 15697, with an average value of 1,927.17± 114.61 CDSs per genome (Table S1). Notably, the chromosomes belonging to the *B. longum* subsp. *infantis* subspecies emerged as the largest ones among the assessed *B. longum* genomes, ranging in size between 2.6 and 2.8 Mb (ANOVA *P*-value <0.05). These results showed that genome size might vary considerably even in closely related strains of the same species, thus indicating remarkable intra-species genetic and physiological diversity, unlike what was previously found for other bifidobacterial species such as *Bifidobacterium bifidum* and *Bifidobacterium dentium* [46, 47].

### Pan-genome and core genome of *B. longum* species

In recent years, computation of the pan genome has been employed as an approach to investigate overall genomic differences and infers the precise phylogenomic relationships between (bifido)bacterial taxa [29, 48–51]. Accordingly, the genomes of *B. longum* strains were subjected to pan-genome analysis, allowing the identification of a total of 22 591 COGs. Analysis of the rate of size increase of the pan genome observed as genomes are sequentially included showed an average of 49.7 newly added COGs at the last three iterations (see Supplementary Material for details). This trend is indicative of a pan genome that has not yet fully reached its completion, though approaching a saturation plateau (Fig. S1). Moreover, a total of 510 COGs were classified as a collection of genes shared by all assessed strains, thereby representing the core genome of the *B. longum* species. Furthermore, the truly unique genes (TUGs) for each *B. longum* strain were also identified, revealing an average of 48.5 TUGs per genome (see Supplementary Material for details). The relatively small number of core genes observed suggests the presence of rather high intra-species variability, particularly when compared to other previously investigated bifidobacterial species, such as *B. bifidum*, *Bifidobacterium breve* (1295 and 1307 conserved COGs, respectively) [46, 52]. On the other hand, the relatively small number of TUGs is comparable with that previously observed for the genomes of *Bifidobacterium pseudolongum* and *B. dentium* (41 and 60 average TUGs, respectively) [29, 47], implying that a large part of the genetic diversity resides in the dispensable gene pool, i.e. those genes that are shared by a subgroup of strains, possibly due to adaptation to specific ecological niches/hosts. Interestingly, *B. pseudolongum* species, for which the subspecies *pseudolongum* and *globosum* are recognized, showed a much larger number of core genes, i.e. 1069 COGs, when compared to those identified in *B. longum* genomes. Therefore, these findings suggest that the latter taxon is characterized by a relatively high intra-specific variability, which may be imputed to distinct genetic traits possessed by each *B. longum* subspecies.

### Phylogenetic analyses the *B. longum* taxon

The pairwise percentage ANI is currently considered to represent the gold standard for inference of close phylogenetic relationships and (sub)species classification of bacterial genomes [40]. Evaluation of the overall genomic differences between the 271 *B. longum* genomes through ANI analysis resulted in values ranging from 94.2 to 98.9 % (Table S2). Notably, previous *Bifidobacterium* phylogenomic studies showed that an ANI threshold value of 94 % properly discriminates between bifidobacterial species [51, 53], being consistent with what has been observed for other phylogenetically related taxonomic groups in the Bifidobacteriaceae family, such as *Gardnerella* [54]. Accordingly, the finding that this phylogenomic analysis generated ANI values above 94.2 % indicates that the included genome sequences correctly fall within the boundaries of a single species, i.e. *B. longum*. Nonetheless, based on the ANI matrix (Table S2), it was possible to identify three subgroups corresponding to the three so far recognized subspecies of *B. longum*, within which the observed ANI values ranged from 96.3 to 98.9 % (Table S2). Furthermore, in order to precisely track the phylogenetic relationships between the strains of this species, we computed a phylogenetic tree based on the amino acid sequence alignment of the 510 COGs that constitute the core genome of this species (Fig. S2). Due to the high number of analysed genomes belonging to the *B. longum* subsp. *longum* subspecies, we decided to generate an additional tree encompassing a pool of 42 representative genomes of this taxon, chosen to maximize the genetic diversity coverage, in order to obtain a clearer graphical visualization of the complete *B. longum* phylogeny (Fig. 1) (see Supplementary Material for details). As expected, the resulting *B. longum*-based phylogenetic tree revealed the presence of three main clades (Figs 1 and S2), consisting of the *B. longum* subsp. *longum* taxonomic group (*Bll*), the *B. longum* subsp. *infantis* taxonomic group (*Bli*) and the *B. longum* subsp. *suis* (*Bls*) taxonomic group (Fig. 1). In-depth analysis of the tree revealed that strains *B. longum* subsp. *infantis* 157F [55], *B. longum* subsp. *infantis* CCUG 52486 [56] and *B. longum* subsp. *longum* JDM301 [57] had been misclassified. Specifically, consistent with what had previously been observed through ANI analysis (Table S2), strains 157F and CCUG 52486 had been assigned to the *B. longum* subsp. *longum* subspecies, while JDM301 had been classified as a member of the *B. longum* subsp. *suis* subspecies. Interpretation of the phylogenomic tree suggests a clear phylogenetic separation between members of *B. longum* subsp. *infantis* cluster and the other *B. longum* strains, indicative of earlier speciation with respect to *B. longum* subsp. *longum* and *B. longum* subsp. suis*,* which showed a closer phylogenetic relationship (Figs 1 and S2, Table S2).

Moreover, the phylogenomic-based approach, combined with ANI value assignment, was applied to taxonomically classify the 11 newly isolated *B. longum* strains in order to include them in subspecies-specific analyses (see below). Specifically, three genomes were shown to belong to *B. longum* subsp. *suis* subspecies, i.e. 209B, 2015B and 2074B, while the remaining eight were classified as members *B. longum* subsp. *longum* subspecies (Fig. 1). Interestingly, *B. longum* subsp. *longum* 1897B, which had been isolated from human milk, was shown to belong to a separate branch with respect to all other *B. longum* subsp. *longum* strains (Fig. 1), denoting a different evolutionary history compared to the other assessed *B. longum* members isolated from the mammalian gut.

### The pan and core- genome of the *B. longum* subspecies

Evolutionary processes have shaped bacterial genomes by driving changes in their genetic repertoire in order to facilitate adaptation to a specific environmental niche [58, 59], thus leading to (sub)speciation events. Pan-genome reconstruction may provide insights into these evolutionary events by unveiling genomic peculiarities and shared genetic traits that characterize a given bacterial taxon [60]. In the context of a *B. longum* subspecies-focused comparative analysis, we separately analysed subspecies-specific pan genomes (Fig. S3) (see Supplementary Material for details). The 251 *B. longum* subsp. *longum* genomes and the ten members of *B. longum* subsp. *suis* used in these analyses showed similar average genome sizes, i.e. of 2.39 and 2.43 Mb (Table S1). The latter are significantly smaller compared to that observed for *B. longum* subsp. *infantis* (average of 2.65 Mb) (Table S1), which also showed an average of 253 additional CDSs when compared to those found in *B. longum* subsp. *longum* and *B. longum* subsp. *suis* genomes (ANOVA *P*-value <0.001) (details in Supplementary Material). This finding suggests that members of the *B. longum* subsp. *infantis* taxon may have evolved as a result of progressive acquisition of new genetic features [58]. The subspecies-specific pan-genome analyses also allowed the definition of the *Bll*-, *Bls*- and *Bli*-core genome (CG), intended as the subspecies-specific core-genes' repertoire. In detail, these subspecies-specific core genomes were defined by taking into account those COGs shared by at least 85 % of the strains belonging to a given *B. longum* subspecies while being absent in the other two subspecies. The decision to consider an 85 % gene-sharing level, rather than the typically employed 100 %, was motivated by the presence of a high number of draft genomes within the analysed genome collection, which therefore could influence the accuracy of the calculation of subspecies-specific core genomes. In this manner, a total of 24 and five core genes represented the *Bll*-CG and *Bls*-CG, respectively, whereas 53 genes were identified as constituting *Bli*-CG (Fig. S3) (details are reported in Supplementary Material). The relatively small size of the *Bll*-CG and *Bls*-CG may, at least in part, be due to their close phylogenetic relationship and to the high number of analysed *Bll* genomes. However, it suggests that the evolutionary path taken by these subspecies may not have led to the acquisition of a substantial number of subspecies-specific competencies compared to their common *B. longum* ancestor. In contrast, the higher number of genes constituting *Bli*-CG suggests that this subspecies was subject to a higher evolutionary pressure that instigated the acquisition of novel genetic traits. Interestingly, 31 (58 %) of *Bli*-CG, 17 (71 %) of *Bll*-CG and four of the five (80 %) of *Bls*-CG were found in other bifidobacterial species with identity >50 % and coverage >80 % by blastp search in currently available bifidobacterial genomes (Table S3). These data suggest, at first glance, that a subgroup of subspecies-specific core genes may have been acquired by a common bifidobacterial ancestor (as indicated by presence in other bifidobacteria) and subsequently lost at subspecies level. Nevertheless, each subspecies seems to have independently acquired new genetic features, with *B. longum* subsp. *infantis* showing the highest number of genes acquired by presumed HGT events (18.8 % of the *Bli*-CG) (Table 1).

**Table 1.** *B. longum* subspecies-specific core genes

***B. longumsubsp.longum***						
**Core gene**	**Prevalence across the subspecies**	**Function**		**Transporter classification database**		**HGT events**
		Interpro Database	Refseq Database	Function	Family	
B1_0665	99 %	Selenoprotein, putative	YbdD/YjiX family protein			Native
B1_0666	98 %	5TM C-terminal transporter carbon starvation CstA	Carbon starvation protein A	Peptide Transporter Carbon Starvation CstA (CstA) Family	2.A.114.-	Native
B1_1343	98 %	Protein of unknown function (DUF3073)	DUF3073 domain-containing protein			Native
B1_0094	98 %	NADH Oxidase	Nitroreductase			Native
B1_0106	98 %	Periplasmic binding protein-like II	Extracellular solute-binding protein			Native
B1_0884	98 %	–	Aldo/keto reductase family protein			Native
B1_1277	97 %	Glycosidases	Pullulanase type I			Native
B1_0628	97 %	l,d-transpeptidase YCIB-related	l,d-transpeptidase			Native
B1_0345	97 %	Metal-dependent hydrolase	Amidohydrolase family protein			Native
B1_1278	97 %		Alpha-amylase			Native
B1_0156	95 %	–	DUF2400 domain-containing protein			Native
B1_1275	95 %	ABC transporter permease protein MG189-related	ABC transporter permease subunit	It binds α-(1,6)-linked glucosides and galactosides	3.A.1.1.53	Native
B1_0738	94 %	–	DUF1846 domain-containing protein			Native
B1_1795	93 %	Acyl-CoA N-acyltransferases (Nat)	GNAT family N-acetyltransferase			Native
B1_0431	91 %	Uncharacterized protein conserved in bacteria C-term(DUF2220)	DUF3322 and DUF2220 domain-containing protein			Native
B1_1294	90 %	–	Substrate-binding domain-containing protein			Native
B1_0735	90 %	–	DUF87 domain-containing protein			Foreign
B1_0883	90 %	Transcriptional dual regulator hcar-related	LysR family transcriptional regulator			Native
B1_0737	89 %	Type VII secretion system protein EsaG-like	–			Foreign
134B_0607	88 %	–	DNA/RNA non-specific endonuclease			Native
134B_0472	88 %	MATE_MepA_like	MATE family efflux transporter			Native
B1_1296	88 %	K+potassium transporter	KUP/HAK/KT family potassium transporter			Native
B1_0107	86 %	Glycosidase family 31	Alpha-xylosidase			Native
B1_0786	86%	zinc-ribbon domain	Zinc ribbon domain-containing protein			Native
***B. longum* subsp. *infantis***						
**Core gene**	**Prevalence across the subspecies**	**Function**		**Transporter classification database**		**HGT events**
		**Interpro Database**	**Refseq Database**	**Function**	Family	
ACJ51545.1	100 %	MFS general substrate transporter domains	MFS transporter	Glucose Transporter (GT) Family	2.A.1.68.1	Native
ACJ53225.1	100 %	Tetratricopeptide-like helical domain	DUF4037 domain-containing protein			Native
ACJ52470.1	100 %	Ttransporter solute:sodium symporter family	Sodium/solute symporter	Glucose or galactose:Na +symporter	2.A.1.68.1	Native
ACJ52099.1	100%	Response regulator receiver domain	Response regulator transcription factor			Foreign
ACJ51227.1	100 %	–	–			Native
ACJ52098.1	100 %	Lantibiotic immunity protein Spa1	NisI/SpaI family lantibiotic immunity			Native
ACJ53071.1	100 %	Pyridoxal-phosphate dependent enzyme	Pyridoxal-phosphate dependent enzyme			Native
ACJ51238.1	100 %	High-affinity nickel-transport protein	Nickel/cobalt transporter			Native
ACJ51551.1	100 %	Bacteriocin (Lactococcin_972)	Lactococcin 972 family bacteriocin			Foreign
ACJ51549.1	100 %	Nitrate/nitrite sensor protein narx-related	Histidine kinase			Native
ACJ53072.1	100 %	metallo-dependent hydrolases	Guanine deaminase			Native
ACJ52052.1	100 %	GDSL-like Lipase/Acylhydrolase family	Lipase			Native
ACJ53073.1	100 %	MFS multidrug transporter	MFS transporter	Tet38 tetracycline-resistance protein	2.A.1.3.22	Native
ACJ51151.1	100 %	–	–			Foreign
ACJ53179.1	100 %	RecG, C-terminal domain superfamily	Transcriptional regulator, partial			Native
ACJ52471.1	100 %	–	–			Native
ACJ51149.1	100 %	RelB antitoxin/Antitoxin DinJ	Type II toxin-antitoxin system family			Foreign
ACJ51552.1	100 %	–	–			Native
ACJ53224.1	100 %	–	5'-nucleotidase C-terminal domain			Native
ACJ51550.1	100 %	Response regulatory domain profile.	Response regulator transcription factor			Native
ACJ51553.1	100 %	Nucleoside triphosphate hydrolases	ATP-binding domain-containing protein			Foreign
ACJ52096.1	100 %	Lantibiotic protection ABC transporter permease	Lantibiotic immunity ABC transporter permease	3-component subtilin immunity exporter	3.A.1.124.2	Foreign
ACJ52097.1	100 %	ABC-2 family transporter protein	Lantibiotic immunity permease	CprABC antimicrobial peptide resistance ABC exporter	3.A.1.124.6	Foreign
ACJ51554.1	100 %	–	–			Native
ACJ51425.1	90 %	Antitoxin	–			Foreign
ACJ51673.1	90 %	ABC superfamily metabolite uptake	ABC transporter permease	Putative macrolide-specific efflux system, MacAB	3.A.1.122.16	Native
ACJ53183.1	90 %	Metallophosphoesterase, calcineurin family	Metallophosphoesterase			Native
ACJ53406.1	90 %	Sialidase	Exo-alpha-sialidase			Native
ACJ52932.1	90 %	MFS_MefA_like	MFS transporter	The tetracycline resistance determinant, TetV	2.A.1.21.3	Native
ACJ52100.1	90 %	HAMP domain-containing histidine kinase	–	–		Native
ACJ52095.1	90 %	lantibiotic, protection ABC transporter ATP binding protein	–	–		Foreign
ACJ51226.1	90 %	Protein/nucleic acid deglycase dj-1-related	DJ-1/PfpI family protein			Native
ACJ53416.1	90 %	Beta-lactamase superfamily domain	MBL fold metallo-hydrolase			Native
ACJ53415.1	90 %	PBP2_UgpB	ABC transporter substrate-binding protein	Involved in maltose and maltodextrin uptake	CEF11988.1	Native
ACJ51154.1	90 %	ABC transporter integral membrane type-1	Phosphonate ABC transporter, permease	Putative phosphonate/phosphite/phosphate porter	3.A.1.9.2	Native
ACJ51426.1	90 %	type II toxin-antitoxin system	BrnT family toxin			Native
ACJ51156.1	90 %	ABC transporter-type domain profile	Phosphonate ABC transporter ATP-binding	Putative phosphonate/phosphite/phosphate porter	3.A.1.9.2	Native
ACJ53417.1	90%	Transport system inner membrane component	Carbohydrate ABC transporter permease	ABC transporters for maltose/maltotriose and trehalose	3.A.1.1.23	Native
ACJ51155.1	90 %	phosphonate ABC transporter, permease	ABC transporter, permease protein	Putative phosphonate/phosphite/phosphate porter	3.A.1.9.2	Native
ACJ51158.1	90 %	SIS_RpiR	MurR/RpiR family transcriptional regulator			Native
ACJ51157.1	90 %	Periplasmatic phosphonate-binding protein	ABC transporter substrate-binding protein	Putative phosphonate/phosphite/phosphate porter, PhnDCE	3.A.1.9.2	Native
ACJ51374.1	90 %	MFS_MdtG_SLC18_like	MFS transporter	Copper Uptake Porter	2.A.1.81.-	Native
ACJ51159.1	90 %	HAD-like superfamily	HAD family hydrolase			Native
ACJ51153.1	90 %	5′-Nucleotidase/apyrase	Metallophosphoesterase			Native
ACJ53414.1	90 %	Haloacid dehalogenase-like hydrolase	HAD family hydrolase			Native
ACJ53419.1	90 %	ABC transporter-type domain profile.	ABC transporter ATP-binding protein	Involved in the uptake of pectin oligosaccharides	3.A.1.1.34	Native
ACJ53418.1	90 %	ABC transporter integral membrane type-1	Sugar ABC transporter permease	The fructooligosaccharide porter	3.A.1.1.20	Native
ACJ51575.1	90 %	Glycosidases	Family 20 glycosylhydrolase			Native
ACJ51984.1	90 %	MetI-like	Sugar ABC transporter permease	The xylobiose porter; BxlEFG(K)	3.A.1.1.21	Native
ACJ51567.1	90%	–	Tyrosine-type recombinase/integrase			Foreign
ACJ51985.1	90 %	Maltose transport system permease	ABC transporter permease subunit	N-Acetylglucosamine/N,N'-diacetyl chitobiose porter	3.A.1.1.18	Native
ACJ53244.1	90 %	Duplicated hybrid motif	PTS glucose transporter subunit IIA			Native
ACJ51983.1	90 %	Carbohydrate substrate-binding protein	Carbohydrate ABC transporter	xylobiose porter	3.A.1.1.21	Native
***B. longum* subsp. *suis***						
**Core gene**	**Prevalence across the subspecies**	**Function**		**Transporter classification database**		**HGT events**
		**Interpro Database**	**Refseq Database**	**Function**	Family	
AIF90321.1	100 %	ABC transporter, atp-binding protein	ABC transporter ATP-binding protein	The Macrolide Exporter (MacB) Family	3.A.1.122.-	Native
AIF90322.1	100 %	ABC transporter permease	MacB-like periplasmic core domain	Exports macrolide antibiotics	3.A.1.122.18	Native
SDO34397.1	70 %	Heavy metal transporter	ABC transporter ATP-binding protein			Native
SDO30658.1	70 %	ABC-2 type transporter	FHA domain-containing protein	ABC exporter involved in bacterial competitiveness	3.A.1.105.19	Native
AIF89665.1	70 %	vWA-like	VWA domain-containing protein			Native

### Functional assessment of *B. longum* subspecies-specific core genomes

In order to gain further insight into the physiological characteristics of each *B. longum* subspecies, we investigated the *Bll*-CG, *Bls*-CG and *Bli*-CG from a functional perspective by similarity searches in the NCBI RefSeq nr database [61] and protein domain prediction by InterProScan [62].

**Fig. 2.**
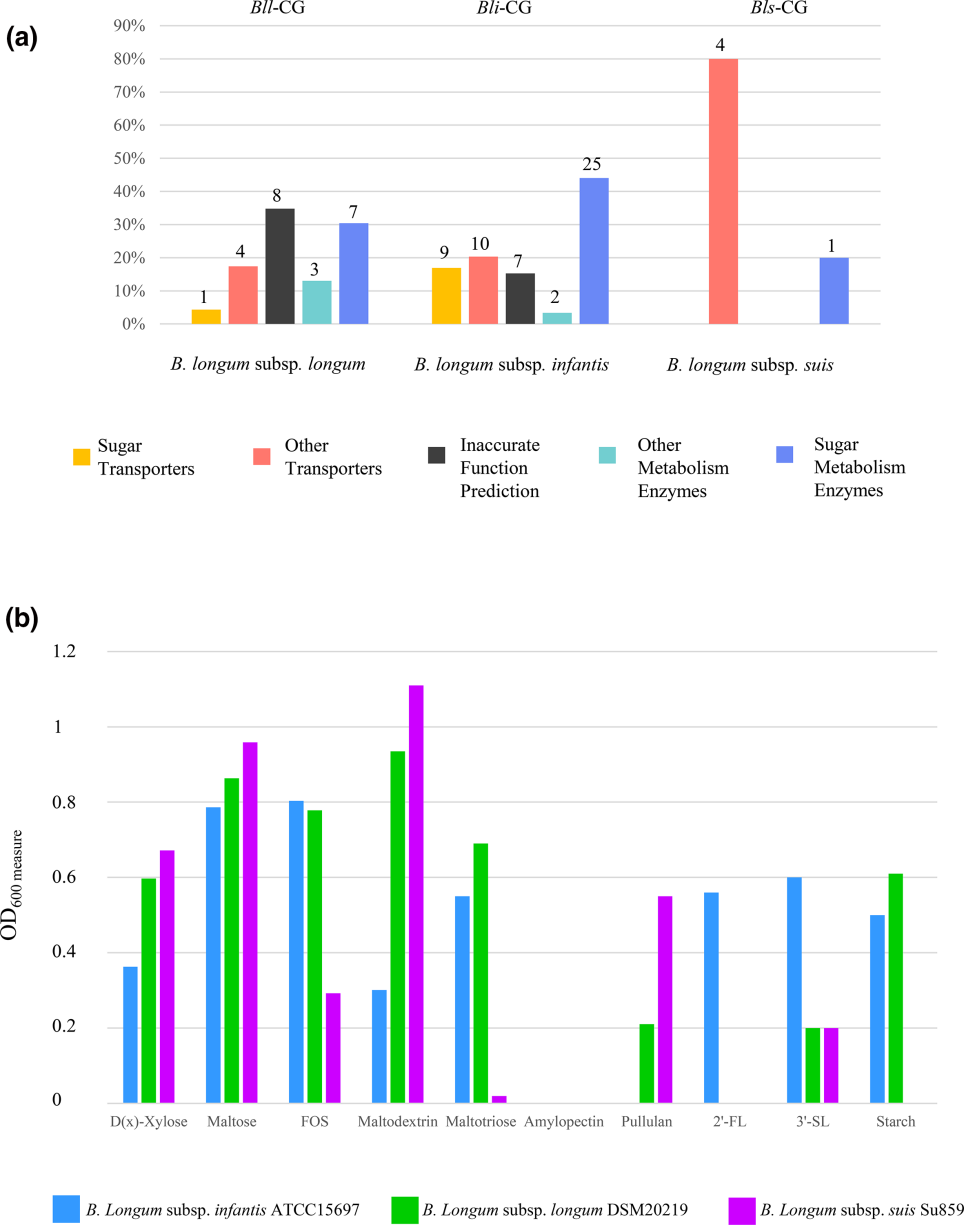
Functional annotation of core genes and growth performance of *B. longum* subspecies. Panel (a) shows the distribution in functional categories of the core genes identified in each *B. longum* subspecies. The number of genes assigned to each category is reported at the top of the columns. Panel (b) displays the growth performance of each *B. longum* type strain on different carbohydrates expressed through measurement of OD_600 nm_.

Of the 24 core genes unique to *B. longum* subsp. longum*,* eight could not be functionally annotated due to the absence of homologues with known function in the RefSeq nr database and known protein domains. In contrast, four were predicted to encode carbohydrate-utilization enzymes (Fig. 2a). Specifically, genes encoding pullulanase type I belonging to glycoside hydrolase family 13 (GH13), alpha-amylase (GH57), and a member of the amidohydrolase family proteins were found to be present in 97 % of the analysed genomes. Moreover, a gene whose protein product resembles members of glycosyl hydrolase family 31, representing enzymes such as alpha-glucosidase, glucoamylase, alpha-xylosidase and sucrase-isomaltase, was detected among 86 % of the analysed *B. longum* subsp. *longum* genomes (Table 1). Interestingly, the above-mentioned enzymes are typically involved in the utilization of plant-related carbohydrates, which, being undigested by the host, are thus available as a carbon source by the microbiota resident in the colon. This finding corresponds with *B. longum* subsp. *longum* being commonly present in faecal samples of human adults, whose diet includes such plant carbohydrates [14].

The functional dissection of genes attributed to *Bls-*CG revealed the ubiquitous presence of two genes encoding ATP-binding cassette (ABC) transporters that are implicated in macrolide resistance. At the same time, among 90 % of the strains, an additional ABC transporter was found to be involved in the detoxification of heavy metals (Table 1, Fig. 2a), allowing us to infer that these transporters play a critical role in niche adaptation of this subspecies. Notably, macrolides have been reported to be among the most frequently used classes of antimicrobials in pig breeding [63]. Therefore, they may have facilitated the development of antimicrobial resistance in bacteria present in the porcine gut microbiota [64].

Focusing on the *B. longum* subsp. *infantis*-specific core genetic repertoire, the most noticeable difference with respect to *B. longum* subsp. *longum* is the presence of genes involved in transporting a broad range of carbohydrates (Fig. 2a). Specifically, all assessed *B. longum* subsp. *infantis* genomes encompass genes that are predicted to encode a glucose transporter and a glucose/galactose-Na^+^ symporter. Interestingly, since glucose and galactose are the building blocks constituting lactose through glycosidic linkage, this finding suggests the presence of an extracellular (bifido)bacterial β-galactosidase (GH42), as also reported in previous genomic surveys [65], and improved specialization toward the uptake of released simple sugars. Moreover, we also identified transporters related to Tetracycline resistance, including the tetracycline resistance determinant Tet(V) and *Tet38* gene. Tetracyclines are one of the most widely used groups of antibiotics worldwide, and resistance to this class of antibiotics is widespread even among bacteria that colonize the infant gut [66, 67]. Therefore, it may represent a trait that increases competitiveness of *B. longum* subsp. *infantis* in the gastrointestinal tract.

Progressively extending the analysis of *Bli*-CG by decreasing the level of gene sharing amongst members of this subspecies, i.e. prevalence, we identified genes that were involved in the uptake of pectic oligosaccharides (POSs), fructooligosaccharides (FOSs), maltose/maltotriose, and xylobiose with a prevalence of 90 % (Table 1). Intriguingly, these observations corroborated the well-known bifidogenic properties exhibited by POSs and FOSs, routinely used in commercial prebiotics due to their beneficial impact on the gut microbiota [68, 69]. Furthermore, within 90 % of *B. longum* subsp. *infantis* genomes, we identified an exo-alpha sialidase (GH33) and a member of glycosyl hydrolase family 20 (GH20) (Table 1), which represent enzymes known to be implicated in the metabolism of HMOs. These latter glycans are not processed by human digestive enzymes, thus reaching the colon intact where they are metabolized by certain members of the resident microbial community, such as *B. bifidum*, *B. breve* as well as *B. longum*, which encode gene clusters specifically dedicated to HMO metabolism [13, 70]. In particular, sialidases (GH33) catalyse the removal of terminal sialic acid residues, thus playing a critical role in the degradation of sialylated HMOs, such as 3′- and 6′-sialyllactose [71]. In line with previous publications, these findings show that degradation of sialylated HMOs is an ability that seems to be distinctive for *B. longum* subsp. *infantis*, thus being a characterizing genotypic and phenotypic feature of this subspecies [15]. In contrast, the above-described GH20 family comprises enzymes with β-hexosaminidase and lacto-N-biosidase activities, which act on substrates that form part of the HMO backbone, thereby releasing N-acetylglucosamine and lactose molecules, respectively [15, 72]. Previous investigations of the bifidobacterial glycobiome have highlighted that most *B. longum* subsp. *longum* strains (75–100 % of the strains) are predicted to encode the β-hexosaminidase (GH20), the lacto-N-biose phosphorylase (GH112), as well as an extracellular lacto-N-biosidase (GH136) [14, 73]. Intriguingly the finding of additional genes belonging to GH20 family encoded only by members of the *B. longum* subsp. *infantis* suggests that this subspecies has been subject to specific evolutionary selection. Remarkably, the latter seems to have driven *B. longum* subsp. *infantis* towards the acquisition of HMO-metabolizing genes, in addition to those shared with other members of the *B. longum* species.

Overall, the observed uneven distribution of the carbohydrate-active enzyme arsenal may reflect the distinct colonization strategy adopted by each *B. longum* subspecies, indicating that *B. longum* subsp. *longum* is more adapted to a (human) adult diet, as also supported by previous findings [15]. In contrast, members of the *B. longum* subsp. *infantis* subspecies may have evolved from a plant-derived glycan utilization gene-makeup towards a genomic repertoire that aims to achieve efficient colonization of the suckling mammalian gut.

To validate these *in silico* results, which indicate a more dedicated commitment of *B. longum* subsp. *longum* toward the breakdown of plant-related carbohydrate when compared to *B. longum* subsp. *infantis*, growth of the type strains of each *B. longum* subspecies, namely *B. longum* subsp. *longum* DSM20219, *B. longum* subsp. *suis* DSM20097 and *B. longum* subsp. *infantis* ATCC15697, was evaluated on ten different carbohydrates. In detail, for growth-profiling experiments, we used a carbohydrate-free basic MRS medium, which was supplemented with either amylopectin, pullulan, starch, maltotriose, maltodextrin, xylose, 2′-FL, 3′-SL, FOS or maltose as the sole carbon source (Table S4, Fig. 2b). Based on our analyses, *B. longum* subsp. *suis* was the only subspecies able to grow on pullulan-based medium (final OD above 0.5). Appreciable growth was also observed on xylose, maltose and maltodextrin (final OD ranging from 0.67 to 1.11). Conversely, both *B. longum* subsp. *longum* and *B. longum* subsp. *infantis* was shown to be able to grow on starch and starch-like glycans (final OD above 0.5), with the exception of amylopectin and pullulan for which no appreciable growth was noticed (Table S4, Fig. 2b). Nevertheless, as is displayed in Fig. 2(b). *longum* subsp. *infantis* was shown to exhibit a reduced level of metabolic abilities on various assessed plant-related glycans when compared to those elicited by *B. longum* subsp. *longum*. Furthermore, *B. longum* subsp. *longum* was shown to possess the most elaborate plant-related carbohydrate degrading activities among the *B. longum* species, being consistent with the above-described *in silico* reports (Table S4, Fig. 2b) [74]. Furthermore, *B. longum* subsp. *infantis* appears to be the only subspecies type strain capable of metabolizing 2′-FL and 3′-SL (Table S4, Fig. 2b). Consistently, the pronounced ability of *B. longum* subsp. *infantis* to metabolize a wide range of HMO compounds has been extensively reported [75–77]. However, carbohydrate metabolism data available in the literature have also highlighted specific HMO-utilizing abilities for certain members of the *B. longum* subsp. *longum*. While all strains can efficiently metabolize lacto-N-tetraose (LNT) and lacto-N-biose (LNB), only certain strains have shown growth capabilities on fucosylated HMOs and Lacto-N-neotetraose (LNnt) [76, 78]. In fact, growth profiles of the latter subspecies resemble that of *Bifidobacterium adolescentis* [74], which represents a gut-resident bifidobacterial taxon typical of the post-weaning period [79].

Overall, the findings related to the *in vitro* growth experiments corroborate our *in silico* data and may be a reflection of the ecological niche in which each *B. longum* subspecies dominate. Our data therefore suggest that *B. longum* subsp. *longum* plays an ecological role in the metabolism of dietary, plant-derived carbohydrates during weaning and post-weaning phases when infants are gradually introduced to a solid diet containing such complex carbohydrates [80–82]). Accordingly, the identified fermentation capabilities may provide an explanation as to how *B. longum* subsp. *longum* is able colonize both the infant and adult gut. In contrast, *B. longum* subsp. *infantis* is more adapted to colonization of the pre-weaning gut environment due to its particular HMO degradation abilities [15, 83].

### Mobilome prediction in *B. longum* genomes

HGT is the process by which genetic material is exchanged between and within microbial taxa/taxon [84, 85]. This phenomenon of acquisition of new genomic properties is crucial for adaptation to new ecological niches [86], while it generates genetic diversity across bacterial taxa [87]. To a large degree, among (bifido)bacteria, HGT is assumed to occur through mobile genetic elements, such as plasmids, transposons or bacteriophages, with the latter considered one of the main vectors for gene transfer [88, 89]. To explore the possibility that HGT events are responsible for the substantial intra-specific genomic diversity observed between *B. longum* subspecies, the genomes of the representative 42 strains previously selected for phylogenetic analyses (Fig. 1) were screened using the software Colombo [41].

**Fig. 3.**
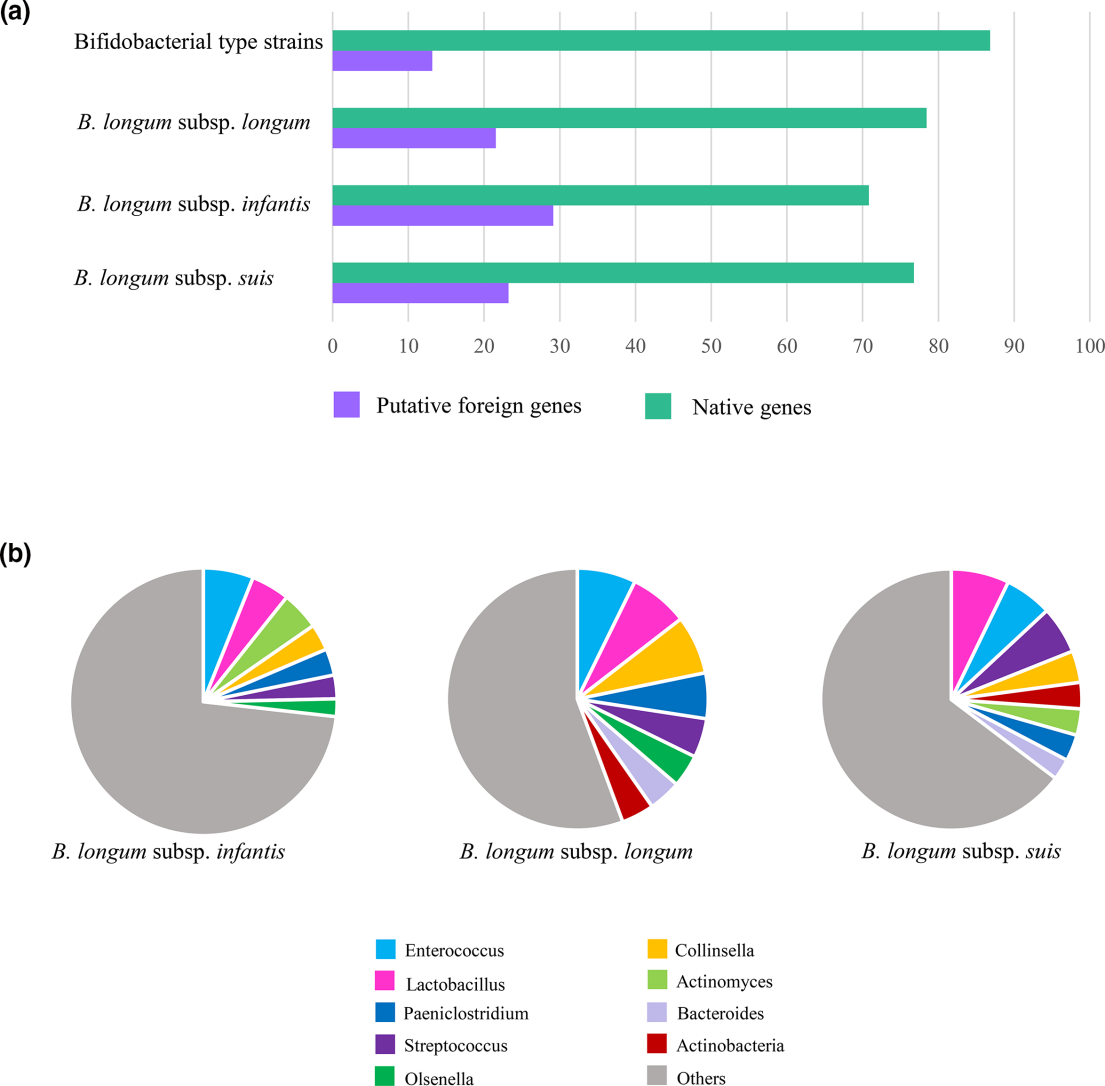
Prediction of *B. longum* subspecies HGT events. Panel (a) shows the average percentages of the predicted foreign genes in each *B. longum* subspecies, compared with those obtained from 85 type strains belonging to different bifidobacterial species. Panel (b) displays the predominant non-bifidobacterial donor genera of the putative alien genes found in each *B. longum* subspecies.

Following bioinformatic inspection of the *B. longum* subsp. *longum* and *B. longum* subsp. *suis* genomes, an average of 431 and 407 putative HGT genes, corresponding to an average of 22.5 and 20.9 % of the total number of CDS, respectively, were identified (Fig. 3a, Table S5). In contrast, an average of 640 CDS, corresponding to 29.5 % of the total number of predicted CDS, were identified as being potentially acquired by HGT in *B. longum* subsp. *infantis* (Fig. 3a, Table S5). To get an idea of the extent to which HGT events have contributed to shaping the genome architecture of *B. longum* subspecies, these values were compared to those obtained from 85 type strains belonging to different bifidobacterial species. Overall, the latter genomes showed an average of 12.8 % putative HGT-acquired genes, which was significantly lower than those identified in *B. longum* subspecies (ANOVA *P*-value <0.01) (Fig. 3a, Table S6), as was previously reported [90]. Furthermore, it is particularly noteworthy that *B. longum* subsp. *infantis* elicits the highest HGT gene numbers among the assessed bifidobacterial (sub)species, highlighting that this subspecies appears to be more suitable or to have been subject to higher selective pressure to acquire alien DNA when compared to not only other bifidobacterial species, but also compared to other *B. longum* subspecies (ANOVA *P*-value <0.01). Accordingly, these results provide an explanation for the higher average genome size of the *B. longum* subsp. *infantis* chromosomes.

Subsequently, the genes predicted to have been horizontally acquired by each of the *B. longum* subspecies were subjected to similarity searches in the NCBI refseq nr database in order to obtain an overview of the potential donor taxa. In particular, 124 (35 %) of the identified foreign genes of *B. longum* subsp. *longum*, 153 (30 %) of those of *B. longum* subsp. *suis*, and 280 (44 %) of the alien genes detected in *B. longum* subsp. *infantis* returned significant database hits in terms of similarity. Interestingly, of these identified HGT genes, 118 (95 %) of *B. longum* subsp. *longum*, 124 (81 %) of *B. longum* subsp. suis*,* and 191 (68 %) of *B. longum* subsp. *infantis*, corresponding respectively to 27, 30, and 30 % of the total HGT-acquired genes, appear to be derived from other bifidobacterial species, most frequently by *B. bifidum*, *B. breve* and *Bifidobacterium adolescentis* (Tables S7–S9). These latter species are also commonly found in the gastrointestinal tract of infants, thus representing a common niche that would facilitate horizontal transfer events. Furthermore, following the exclusion of hits corresponding to genera belonging to the Bifidobacteriaceae family, the analysis revealed a preferential origin of alien DNA from *Enterococcus*, *Lactobacillus*, *Streptococcus*, *Collinsella*, *Bacteroides, Actinomyces* as well as *Paeniclostridium* (Tables S7–S9). In particular, *Enterococcus* (7.2 %), *Lactobacillus* (7.2 %), and *Collinsella* (7.2 %) were identified as major donors of the *B. longum* subsp. *longum* horizontal genes (Fig. 3b, Table S8), while *Lactobacillus* (7.1 %)*, Enterococcus* (5.8 %), and *Streptococcus* (5.8 %) were recognized as the prominent donors of the *B. longum* subsp. *suis* foreign genes (Fig. 3b, Table S9). In a similar fashion, the *B. longum* subsp. *infantis* genes putatively acquired by HGT were predicted to be originated mainly from *Enterococcus* (6.1 %), *Lactobacillus* (4.6 %) and *Actinomyces* (4.6 %) (Fig. 3b, Table S7). Interestingly, these donor genera, including the bifidobacterial ones, are known to share the human (infant) gut environment with members of *B. longum* species [7, 91], thus providing the opportunity for genetic transfer events, which can act as the driver of niche adaptation in members of the *B. longum* species [92].

To further investigate how HGT can contribute to differentially shape the *B. longum* subspecies, we assessed to what extent potential HGT events affect the specific core genome of each *B. longum* subspecies (Table 1). Notably, we found two alien core genes in the *Bll*-CG, and ten putative alien genes in the *Bli*-CG, corresponding respectively to 8.3 and 18.8 % of their own total number of core genes. Instead, no horizontal core genes were found among the five constituting the *Bls*-CG (Table 1). As expected, HGT seems to contribute only marginally to the core genome of the three *B. longum* subspecies. This observation is consistent with the notion that core genes are the most ancient genes, whose acquisition shaped the ancestors of each *B. longum* subspecies [93, 94]. Nevertheless, *B. longum* subsp. *infantis* was predicted to possess a higher number of foreign core genes compared to the other *B. longum* subspecies. Furthermore, based on RefSeq database annotation, horizontally acquired core genes in *Bli*-CG encompassed five genes putatively involved in the production of antimicrobial peptides, such as bacteriocins, and genes related to a toxin/antitoxin system (Table 1). Notably, bacteriocins are commonly produced by lactic acid bacteria, including *Lactobacillus*, *Streptococcus* and members of the *Enterococcus* genus [95, 96] that were consistently found amongst the major donor genera of foreign *B. longum* genes.

### Survey of genetic features supporting HGT events

**Fig. 4.**
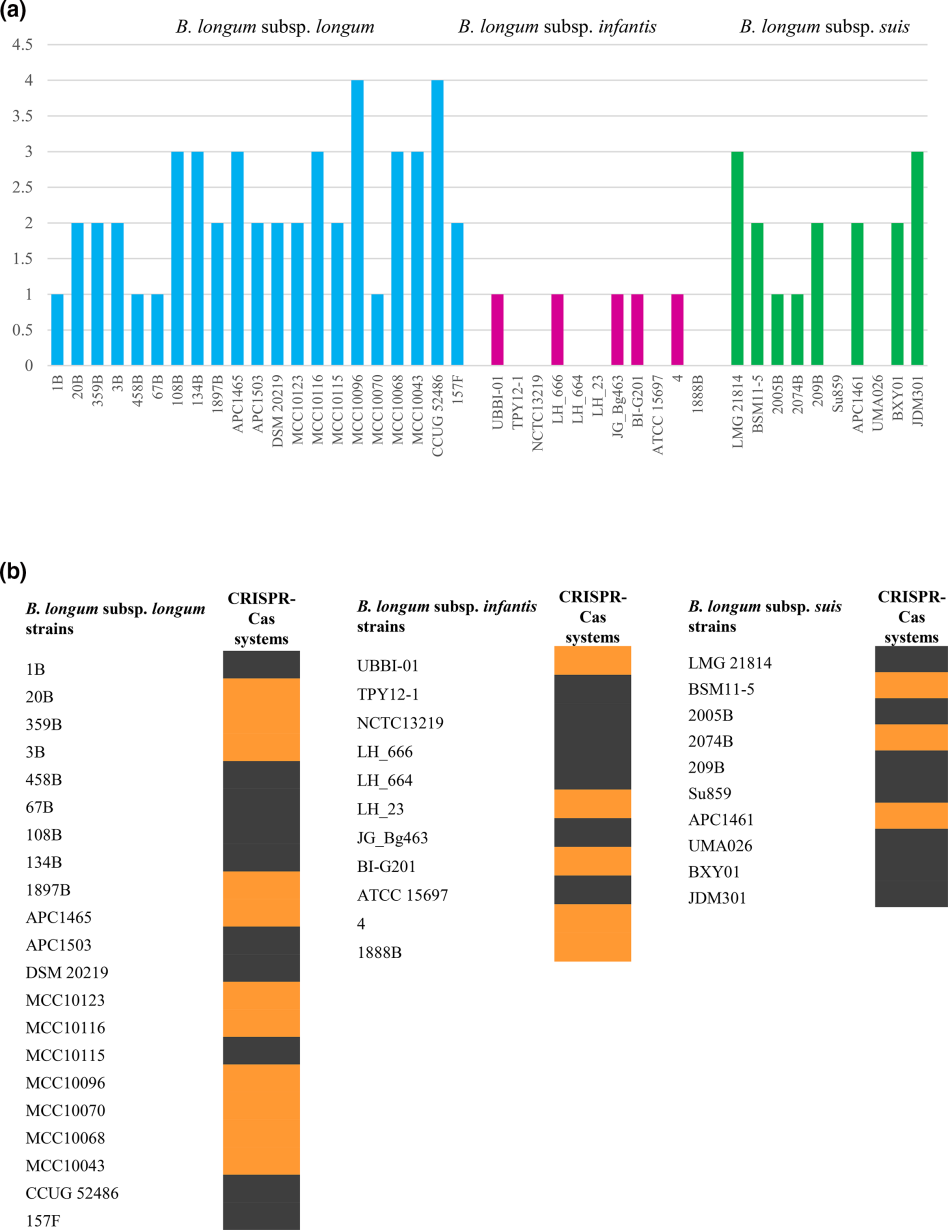
R-M and CRISPR-Cas systems in *B. longum* subspecies. Panel (a) shows the number of genomic R-M systems found in each of the 58 representative *B. longum* strains. Panel (b) depicts the presence (orange) or absence (black) of CRISPR-Cas systems in each of the 58 representative *B. longum* strains.

Mobile genetic elements, such as transposable elements and prophage-like elements, can promote DNA acquisition and facilitate the genetic material transmission between different bacterial taxa [87]. Conversely, CRISPR and R-M systems, which both represent microbial defence mechanisms against invasion of alien genetic material, are responsible for the degradation of nonself-DNA thereby preventing HGT events [97]. In order to investigate the genetic features of *B. longum* subspecies involved in the acquisition of foreign DNA, the representative 42 genomes were screened for R-M and CRISPR-Cas systems (Fig. 4a, b, Table S10). Overall, these analyses revealed that *B. longum* genomes mainly harbour type II and type I R-M systems, with a higher average number of R-M enzymes found in the subspecies *B. longum* subsp. *longum*. In detail, this latter subspecies exhibited an average of 2.2± 0.8 R-M genes (Fig. 4a, Table S10), while assessments of *B. longum* subsp. *suis* and *B. longum* subsp. *infantis* revealed the presence of 1.7± 1.1 and 0.45± 0.5 R-M genes, respectively (Fig. 4a, Table S9). Interestingly, these results negatively correlate with the number of alien genes as mentioned above for each *B. longum* subspecies (*t*-test *P*-value <0.05), corroborating the hypothesis that R-M systems counteract HGT events. As mentioned above, CRISPR-Cas systems represent another bacterial defence mechanism against invading alien DNA [98]. Based on our screening, out of the 21 representative *B. longum* subsp. *longum* strains considered, 11 were shown to contain at least one complete CRISPR-Cas locus in their genome (prevalence of 52.4 %) (Fig. 4b, Table S10). Besides, complete CRISPR-Cas systems were detected in 3 out of the 10 *B. longum* subsp. *suis* genomes*,* corresponding to a prevalence of 30 %, as well as within 3 out of the 11 *B. longum* subsp. *infantis* chromosomes, corresponding to a prevalence of 27 % (Fig. 4b, Table S10). Furthermore, the screening highlighted the occurrence of type I (subtypes I-C, I-E and I-U) and type II systems (subtypes II-C), characterized by the presence of *cas3* and *cas9* genes, respectively [99]. Specifically, a type II CRISPR-Cas system was detected only among *B. longum* subsp. *longum* strains, while such a system seems to be absent in *B. longum* subsp. *suis* and *B. longum* subsp. *infantis*. Overall, profiling of defence mechanisms highlighted that *B. longum* subsp. *longum* genomes seem to be equipped with a more efficient defence against foreign DNA invasion compared to those of both *B. longum* subsp. *suis* and *B. longum* subsp. *infantis* [100].

To obtain an overview of the *B. longum* genetic elements that may be implicated in HGT events, we screened for the presence of prophage-like and IS elements as well as plasmid sequences (Table S10). This allowed the identification of 21 (average of 1 per genome) and 8 (average of 0.8 per genome) prophage-like sequences in the inspected *B. longum* subsp. *longum* and *B. longum* subsp. *suis* genomes, respectively. In contrast, 22 (bifido)prophage, corresponding to an average of 2 integrated phages per genome, were observed in the chromosomes of *B. longum* subsp. *infantis* (Table S10). The genomic structural features of these retrieved bifidoprophages suggest that they represent members of the *Siphoviridae* family, consisting of lysogeny, DNA replication, DNA packaging, head and tail synthesis, and host lysis modules (Table S11). Furthermore, on average, 26.3±11.8 and 23.4±8.8 transposase genes per genome were found by inspecting *B. longum* subsp. *longum* and *B. longum* subsp. *suis* genomes, respectively, while *B. longum* subsp. *infantis* harbours 24.2±19.9 transposase genes per genome. In contrast, *in silico* prediction did not reveal any plasmid sequences among the inspected *B. longum* genomes.

Altogether, these results, coupled with data obtained from the analysis of HGT occurrences, suggests that *B. longum* subsp. *infantis* seems to be more prone to acquire alien genes than the other two *B. longum* subspecies, highlighting how HGT events may represent one of the key factors that shaped the genome of this taxon, thus contributing, to some extent, to provide it with specific ecological niche adaptations.

## Conclusions

We investigated the genome diversity of *B. longum* species and its subspecies *B. longum* subsp. longum*, B. longum subsp. *infantis** and *B. longum* subsp. *suis* through comparative genomic analyses and phylogenomic reconstruction of 261 publicly available and high-quality genomes, along with 11 novel strains sequenced as part of this study. These analyses revealed that members of *B. longum* subsp. *infantis* appear to contain a more extensive genetic repertoire than the other *B. longum* strains, highlighting how the former was shaped over the course of evolution through the acquisition of new genetic features. Notably, the functional analyses of the core genome unveiled that members of *B. longum* subsp. *infantis* possess unique carbohydrate utilization capabilities toward host glycans, particularly those for HMO degradation. When we investigated to what extent HGT events had been responsible for shaping *B. longum* subsp. *infantis* genomes, we revealed the increased frequency by which *B. longum* subsp. *infantis* had acquired alien DNA when compared to the other *B. longum* subspecies and to the type strains of other known bifidobacterial species. Notably, such higher genome plasticity, supported by specific genetic features such as lower number of restriction/modification and CRISPR-Cas systems coupled with a higher occurrence of prophage-like elements, appears to be a possible factor that allowed *B. longum* subsp. *infantis* to adapt to early life mammalian gut colonization. Furthermore, prediction of putative donor taxa of alien DNA revealed a preferential origin from other bifidobacterial and non-bifidobacterial species inhabiting the gut environment, suggesting that the extensive milk-related carbohydrate utilization capabilities that characterize the *B. longum* subsp. *infantis* subspecies may have been obtained through extensive gene harvesting from co-colonizing bacterial taxa. Though our findings provide insights into how the three *B. longum* subspecies probably developed at least in part through differential gene acquisition and subsequent niche occupation, it should be kept in mind that our conclusions are predominantly based on bioinformatic analyses. Our future efforts will therefore aim to further support these *in silico* data with experimental evidence.

Nevertheless, certain limitations of this study should be kept in mind. In particular, the fact that the number of publicly available sequenced chromosomes belonging to *B. longum* subsp. *infantis* and *B. longum* subsp. *suis* is significantly lower compared with that of *B. longum* subsp. *longum* subspecies. This may imply that the genetic variation within the first two subspecies may not have been completely disentangled, as also demonstrated by the identification of an open pan genome characterizing *B. longum* subsp. *infantis* as well as *B. longum* subsp. *suis* (see Supplementary Material). In addition, our study very much focused on the *in silico* assessment of the genetic traits distinguishing each *B. longum* subspecies, highlighting the need for experimental validation of our presented bioinformatics data.

## Supplementary Data

Supplementary material 1

Supplementary material 2
