# Supplementary material for: Phylogenomic disentangling of the Bifidobacterium longum subsp. infantis taxon
Source: Microb Genom. 2021 Jul 28;7(7):000609. doi: 10.1099/mgen.0.000609 (PMC8477406; doi:10.1099/mgen.0.000609)
Supplement: Supplementary material 1 [file mgen-7-0609-s001.pdf]

Phylogenomic disentangling of the *Bifidobacterium longum* subsp. *Infantis* taxon

Running title: Phylogenomic dissection of Bifidobacterium longum

Key words: bifidobacteria, comparative genomics, longum, infantis, HGT

Chiara Tarracchini[1], Christian Milani[1,2], Gabriele Andrea Lugli[1], Leonardo Mancabelli[1], Federico Fontana[1,3], Giulia Alessandri[1], Giulia Longhi1,[3], Rosaria Anzalone[3], Alice Viappiani[3], Francesca Turroni[1,2] Douwe van Sinderen[4], and Marco Ventura[1,2]

Laboratory of Probiogenomics, Department of Chemistry, Life Sciences, and Environmental Sustainability, University of Parma, Parma, Italy[1]; Microbiome Research Hub, University of Parma, Parma, Italy[2]; GenProbio srl, Parma, Italy[3]; APC Microbiome Ireland and School of Microbiology, Bioscience Institute, National University of Ireland, Cork, Ireland[4]

Correspondence. Mailing address for Marco Ventura Laboratory of Probiogenomics, Department of Chemistry, Life Sciences, and Environmental Sustainability, University of Parma, Parco Area delle Scienze 11a, 43124 Parma, Italy. Phone: ++39-521-905666. Fax: ++39-521-905604. E-mail: marco.ventura@unipr.it

Supplementary Text

Pan-genome and core genome of *B. longum* species. Recently, pan-genome computation has allowed to investigate genomic differences between a given (bifido)bacterial taxa as well as their evolutionary development and phylogenomic relationships (1-3). In the framework of a species-level genomic comparative analysis, the available genomes of *B. longum* were sent to pan-genome analysis, revealing a total of 22.591 Clusters of Orthologous Groups (COGs), defined as CDSs showing identity > 50% with alignment coverage > 80%. The pan-genome curve was built plotting the total number of COGs as a function of the 272 *B. longum* genomes, showing an asymptotic behavior with a growth rate progressively decreasing (Figure S1). More precisely, the number of new genes added by sequential inclusion of genomes started from an average of 413.4 at the first three iterations, decreasing to an average of 49.7 at the last three iterations. This trend is indicative of a pan-genome power trend line that has not yet reached the plateau, suggesting that any additional genome included in the analysis will result in slight increases of the pangenome size.

Moreover, the pan-genome analysis also revealed the core genome of this species as well as the unique genes repertoire of each analyzed strain (Figure S1). A total of 510 COGs of *B. longum* pan-genome were shared by all the strains, thus representing the core genome of this species. The Truly Unique Genes (TUGs) for each *B. longum* strain, i.e., the genes present in just one strain, were also identified, revealing a number of TUGs ranging from 296 genes for *B. longum* subsp. *longum* 1897B to 14 genes for two *B. longum* subsp. *longum* strains, i.e., 9 and MC-42, with an average of 48.5 TUGs per genome. These obtained average number of TUGs resulted comparable with that previously evidenced for *Bifidobacterium pseudolongum*, *Bifidobacterium dentium,* showing an average of 41 and 60 TUGs per genome, respectively (4, 5). These findings, coupled with the prediction of a pan-genome curve tending to a plateau, suggested that the evolutionary pathway undertaken by this species has not led to obtaining a high grade of strain-specific genomic variability, probably because of a high grade of specialization

toward a settlement in a limited range of ecological niches, e.g. mammalian gastrointestinal tract. Nevertheless, the concomitant relatively small number of core genes, revealed a potential high grade of intra-specific genomic variability, compared to that previously observed in members of *Bifidobacterium bifidum* and *Bifidobacterium breve,* showing 1295 and 1307 of COGs, respectively (1, 6).

Phylogenetic analyses the *B. longum* taxon. To further explore the overall genomic variability of *B. longum* genome we observed that the pairwise percent Average Nucleotide Identity (ANI) ranging from 94.2 % to 98.9 (Table S2). Notably, previous *Bifidobacterium* phylogenomic studies showed that an ANI threshold value of 94 % properly discriminates between bifidobacterial species (3, 7) as also reported by literature for other genera belonging to *Bifidobacteriaceae* family (8). Accordingly, the ANI values above 94.1 % observed from this phylogenomic analysis revealed that all genome sequences correctly fall into the same species, i.e., *B. longum*. Nonetheless, based on the ANI matrix encompassing all the 272 genomes (Table S2), it was possible to identify three subgroups corresponding to the three-known subspecies of *B. longum*, within which the observed ANI values ranged from 96.3 % to 98.9 %, with an average value of 98.26% (Table S2). Furthermore, in order to explore the phylogenetic relationships between the strains of this species, we computed a phylogenetic tree based on the aminoacidic sequence alignment of the 510 COGs constituting the core genome of this species (Figure S2). Notably, previous literature showed that this approach allows precise high-resolution reconstruction of the phylogenetic relationship between both distant and closely related taxa/strains (3, 8, 9). Due to the high number of analyzed genomes belonging to the *B. longum* subsp. *longum* subspecies, we decided to generate an additional tree encompassing just a pool of 21 representative genomes of this taxon in order to obtain a better and more clear graphical visualization of the whole *B. longum* species phylogeny (Figure 1). In particular, the selection of *B. longum* subsp. *longum* strains displayed in Figure 1 include the type strain, i.e., DSM20219, as well as 12 genomes that had shown the lowest ANI values within the *B. longum*

subsp. *longum* subgroup respect to the type strain in order to maximize genomic variability among strains selected for this subspecies (Table S2). Moreover, all the 11 strains isolated in the framework of this study were also included in Figure 1. As expected, the resulting *B. longum*-based phylogenetic tree revealed the presence of three main clades (Figure 1; Figure S2). These three clades encompassed respectively 251, 11 and 10 *B. longum* genomes, constituting the *B. longum* subsp. *longum* taxonomic group (*Bll*), the *B. longum* subsp. *infantis* taxonomic group (*Bli*) and the *B. longum* subsp. *suis* (*Bls*) taxonomic group, respectively (Figure 1). An in-depth analysis of the tree revealed that four genomes without subspecies classification as well as one that was presumed to belong to the *B. longum* subsp. *longum* subspecies, i.e., JDM301, clustered in the *Bls* clade, thus suggesting a misclassification of this latter strain (Figure 1; Figure S2), consistently with what previously observed through ANI analysis (Table S2). Likewise, CCUG 52486 and 157F strains fall into *Bll* group, despite being previously classified as *B. longum* subsp. *infantis*, thus indicating their mistaken taxonomic classification, also confirmed by ANI analysis (Table S2). Notably, all the subsequent analyses performed in this study were conducted exploiting taxonomic assignments derived from the phylogenomic and ANI analysis. Interpretation of the phylogenomic tree suggests a clear phylogenetic separation between members of *B. longum* subsp. *infantis* cluster and the other *B. longum* strains, indicative of earlier speciation respect to *B. longum* subsp. *longum* and *B. longum* subsp. *suis,* which showed closer phylogenetic relationship.
Moreover, the phylogenomic-based approach, combined with ANI values assignment, was also exploited to taxonomically classify the 11 newly isolated *B. longum* strains.

The Pan- and Core- genome of the *B. longum* subspecies. Pan-genome reconstruction can contribute to deciphering these evolutionary events, by unveiling the genomic peculiarities as well as the shared genetic traits characterizing a given bacterial taxon (10). In this framework, we separately conducted a subspecies-specific pan-genome analyses, involving the 251 *B. longum* subsp. *longum*, 11 *B. longum*

subsp. *infantis* and ten members of *B. longum* subsp. *suis* (Figure S3), These analyses also lead to the definition of the *Bll*-, *Bls*- and *Bli*-Core Genome (CG) as the set of subspecies-specific core genes. In detail, for building the *Bll*- *Bls*- and *Bli*-CG we take into account those COGs shared by at least 85 % of the strains belonging to the same *B. longum* subspecies, and absent in the others. The *B. longum* subsp. *longum* genomes and the members of *B. longum* subsp. *suis* showed an average genome sizes of 2.39 Mb and 2.43 Mb, corresponding to an average of predicted CDS of 1916 and 1947, respectively (Table S1).Moreover, such genome sizes were significantly reduced than those of members of *B. longum* subsp. *infantis* (average of 2.65 Mb corresponding to 2170 CDS per genome) (Table S1) (ANOVA p-value < 0.001). Thus, these findings suggested that during evolution *B. longum* subsp. *infantis* taxon may have obtained an increase in its genome size by progressive acquisition of new genetic materials (11). Furthermore, analysis of the pangenome curves obtained for the three subspecies revealed that *B. longum* subsp. *longum* tend toward reaching a plateau (Figure S3), as indicated by an average addition of 41.8 genes added to the pangenome in the last three iterations. In contrast, *B. longum* subsp. *suis* and *B. longum* subsp. *infantis* pan-genome showed respectively an average of 191.4 and 99.1 genes added at the last three iterations. These data demonstrated that both *B. longum* subsp. *infantis* and *B. longum* subsp. *suis* were characterized by an open pan-genome (Figure S3), implying that further genomic sequencing efforts will extend our knowledge regarding the genetic variability of these taxa.

Following subspecies-specific core genome investigation, a total of 59 genes was retrieved from *Bli*-CG, while 23 and five core genes represented *Bll*-CG and *Bls*-CG, respectively. Notably, 20 of the 59 genes constituting *Bli*-CG as well as two of the five genes forming *Bls*-CG were found within 100 % of the strains belonging to the respective subspecies (Table 1). In contrast, no gene was found to be shared by all the strains constituting the *B. longum* subsp. *longum* subspecies (Table 1), probably due to the higher number of genomes retrieved for this subspecies, of which the majority were available as draft.

References

1. Lugli GA, Duranti S, Albert K, Mancabelli L, Napoli S, Viappiani A, et al. Unveiling Genomic Diversity among Members of the Species Bifidobacterium pseudolongum, a Widely Distributed Gut Commensal of the Animal Kingdom. Appl Environ Microbiol. 2019;85(8).
2. Duranti S, Milani C, Lugli GA, Mancabelli L, Turroni F, Ferrario C, et al. Evaluation of genetic diversity among strains of the human gut commensal Bifidobacterium adolescentis. Sci Rep. 2016;6:23971.
3. Lugli GA, Milani C, Turroni F, Duranti S, Mancabelli L, Mangifesta M, et al. Comparative genomic and phylogenomic analyses of the Bifidobacteriaceae family. BMC Genomics. 2017;18(1):568.
4. Bottacini F, O'Connell Motherway M, Kuczynski J, O'Connell KJ, Serafini F, Duranti S, et al. Comparative genomics of the Bifidobacterium breve taxon. BMC Genomics. 2014;15:170.
5. Duranti S, Milani C, Lugli GA, Turroni F, Mancabelli L, Sanchez B, et al. Insights from genomes of representatives of the human gut commensal Bifidobacterium bifidum. Environ Microbiol. 2015;17(7):2515-31.
6. Lugli GA, Tarracchini C, Alessandri G, Milani C, Mancabelli L, Turroni F, et al. Decoding the Genomic Variability among Members of the Bifidobacterium dentium Species. Microorganisms. 2020;8(11).
7. Lugli GA, Milani C, Duranti S, Mancabelli L, Mangifesta M, Turroni F, et al. Tracking the Taxonomy of the Genus Bifidobacterium Based on a Phylogenomic Approach. Appl Environ Microbiol. 2018;84(4).
8. Tarracchini C, Lugli GA, Mancabelli L, Milani C, Turroni F, Ventura M. Assessing the Genomic Variability of Gardnerella vaginalis through Comparative Genomic Analyses: Evolutionary and Ecological Implications. Appl Environ Microbiol. 2020;87(1).
9. Milani C, Lugli GA, Duranti S, Turroni F, Bottacini F, Mangifesta M, et al. Genomic encyclopedia of type strains of the genus Bifidobacterium. Appl Environ Microbiol. 2014;80(20):6290-302.
10. Medini D, Donati C, Tettelin H, Masignani V, Rappuoli R. The microbial pan-genome. Curr Opin Genet Dev. 2005;15(6):589-94.
11. Koskiniemi S, Sun S, Berg OG, Andersson DI. Selection-driven gene loss in bacteria. Plos Genet. 2012;8(6):e1002787.

Supplementary figure legends

Figure S1. Pan-genome of *B. longum* species. Panel a shows the pangenome size based on the sequential addition of the 289 *B. longum* genomes. Panel b displays the number of core genes (light red), dispensable genes (light blue), and unique genes (purple) identified in the pangenome analysis.

Figure S2. Complete *B. longum*-based phylogenomic tree. The phylogenomic tree, showing all the 289 genomes of *B. longum* included in this study, was based on the concatenation of the 501 *B. longum* core genes and was built through the neighbor-joining method. Bootstrap percentages above 50 are shown at node points, based on 1,000 replicates. Phylogenetic clusters are highlighted with similarly colored branches.

Figure S3. Prediction of the *B. longum* subspecies pangenome. For each *B. longum* subspecies**,** Panel a shows the pan-genomes curve representing the variation of pan-genome size resulting from the sequential genomes addition. Panel b represents a pie chart of the number of core genes (red), dispensable genes (light blue) and unique genes (purple).

a)

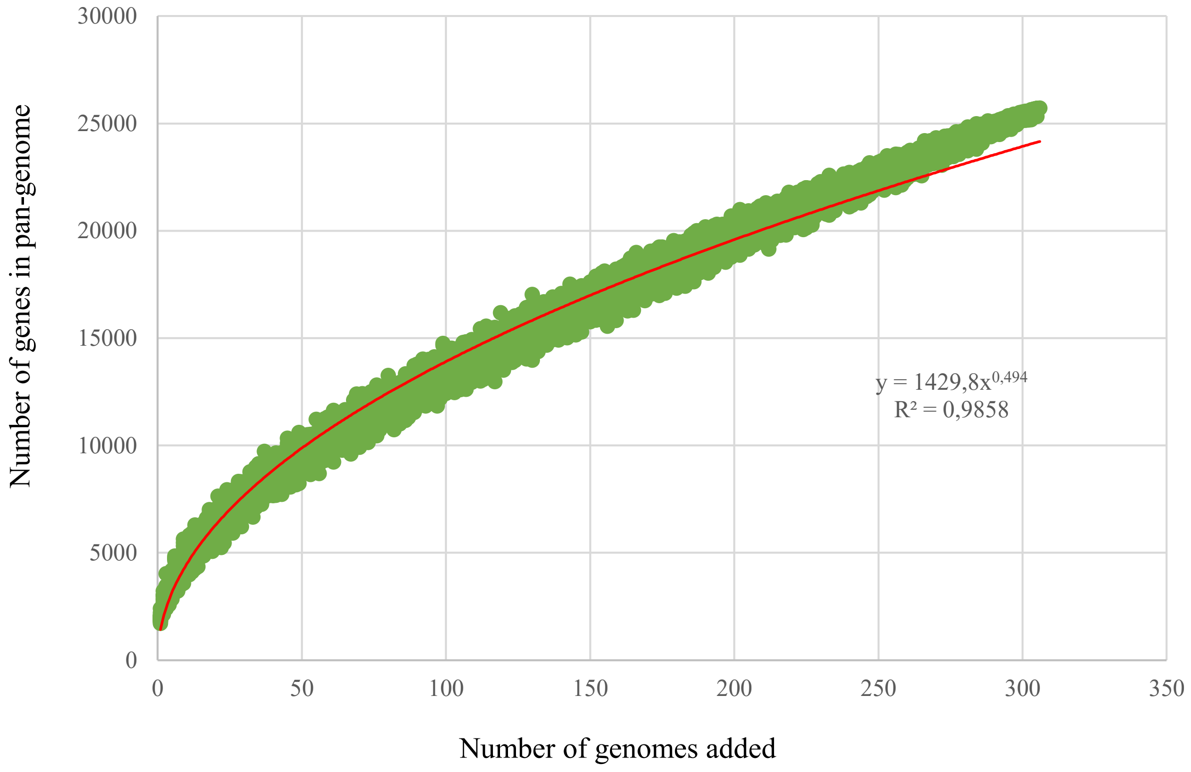
30000
25000
20000
15000
10000
5000
0
Number of genes in pan-genome
0
50
100
150
200
250
300
350
Number of genomes added
y = 1429,8x0,494
R² = 0,9858

b)

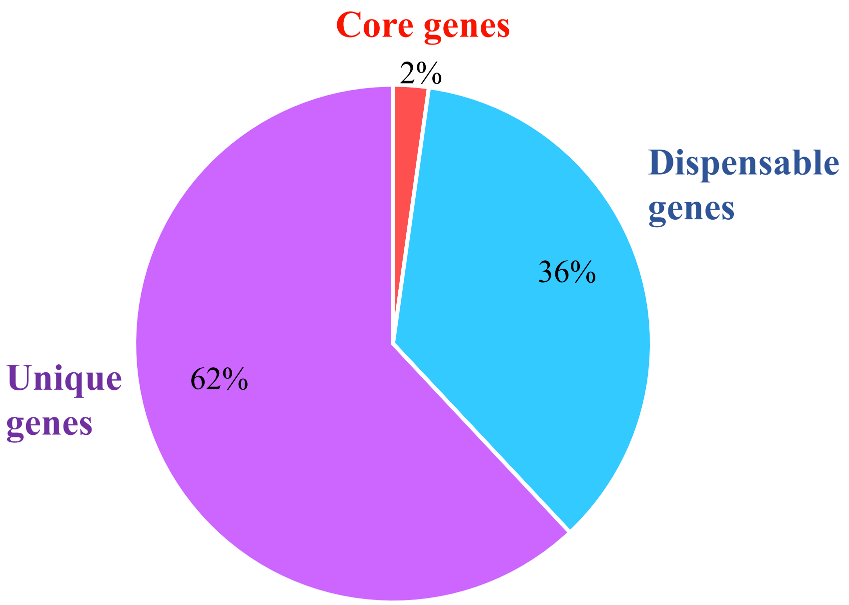
Core genes
2%
Dispensable genes
36%
Unique genes
62%

Figure S1

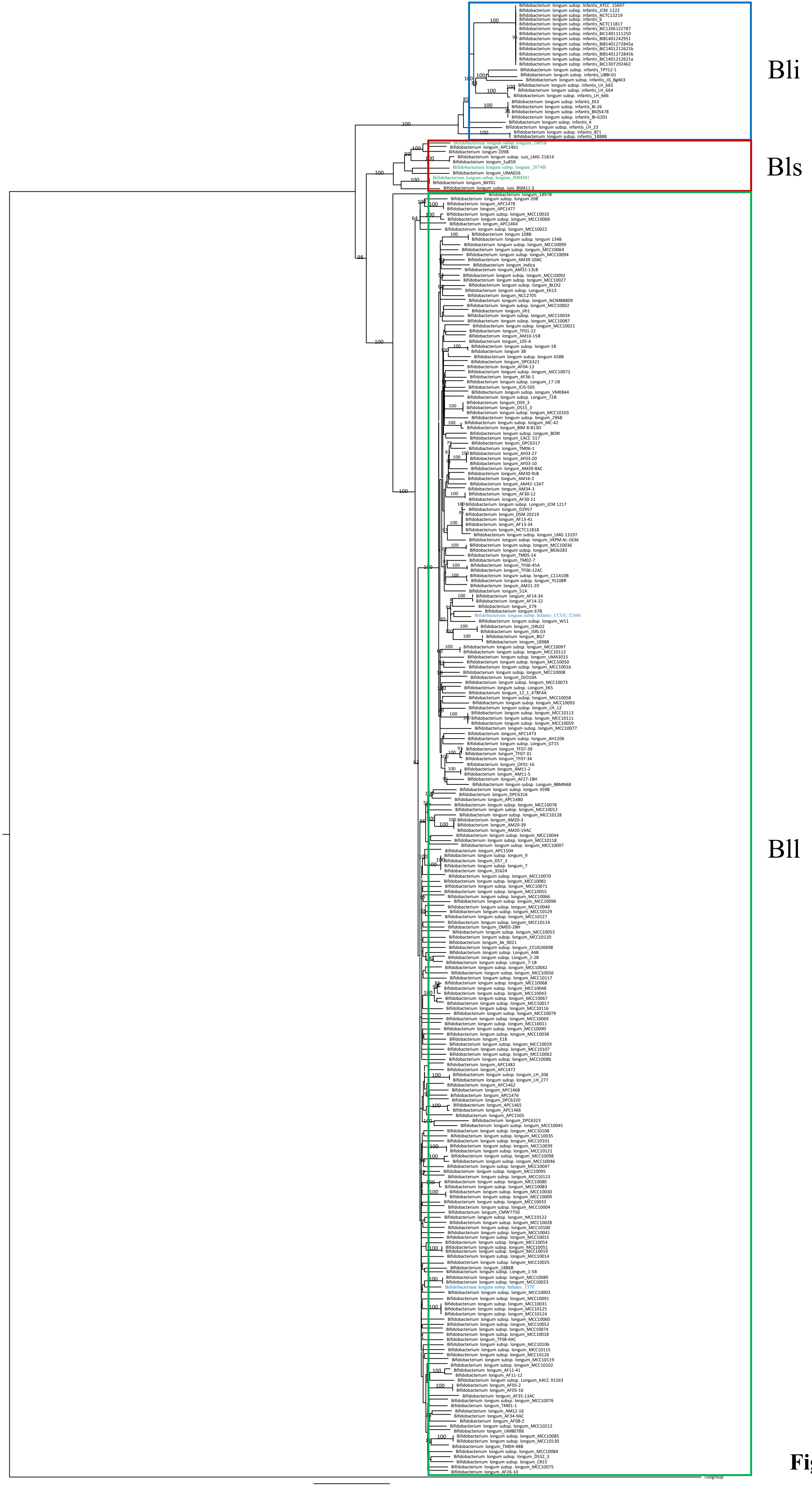

Figure S2

***B. longum* subsp. *infantis* pangenome**

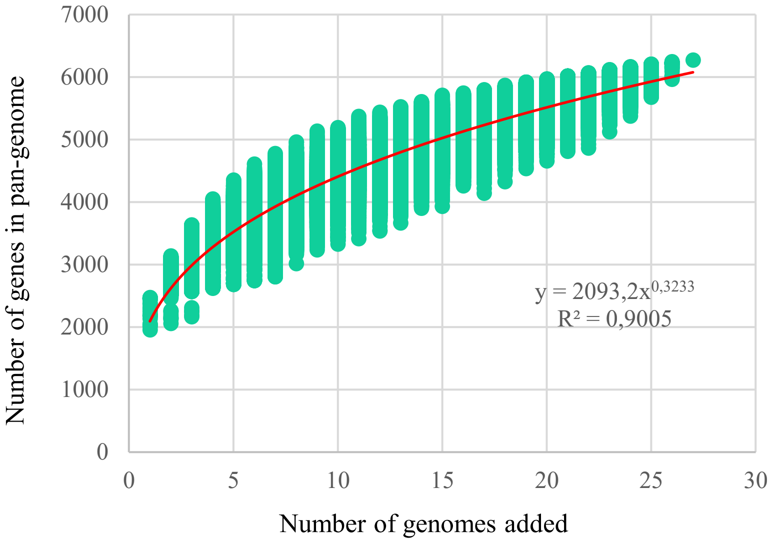

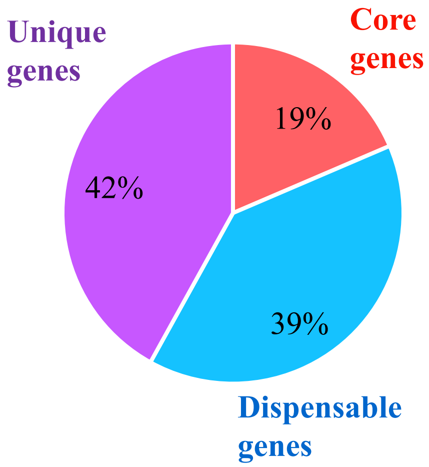

***B. longum* subsp. *suis* pangenome**

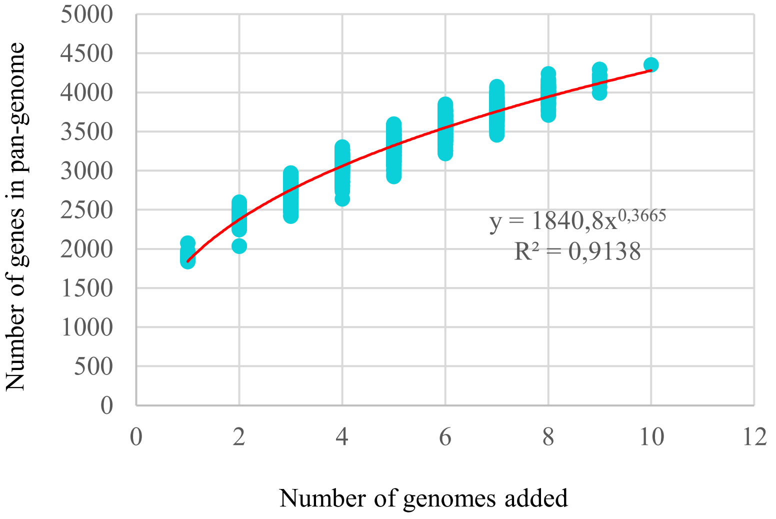

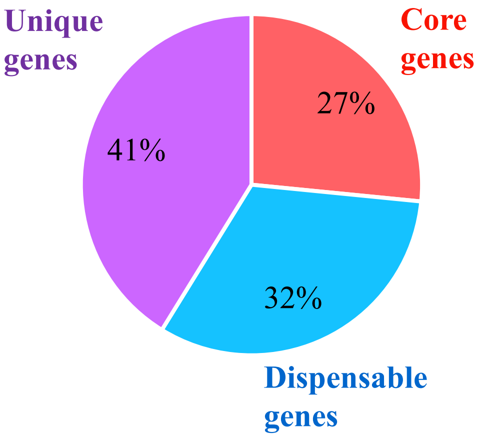

***B. longum* subsp. *longum* pangenome**

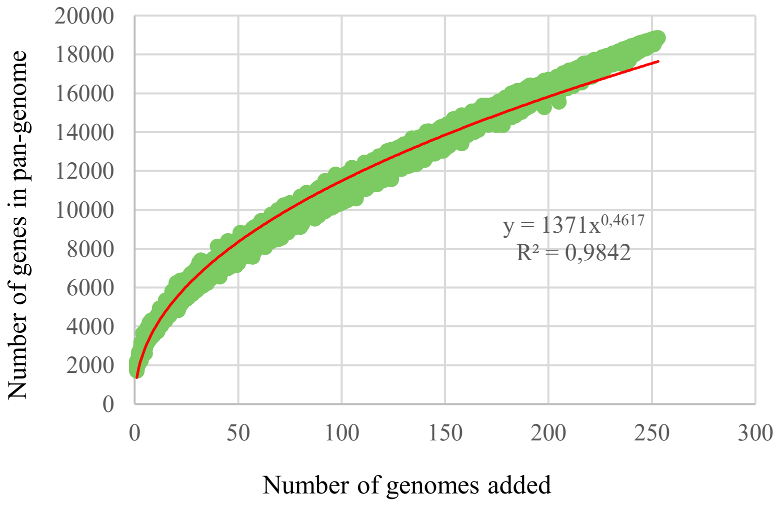

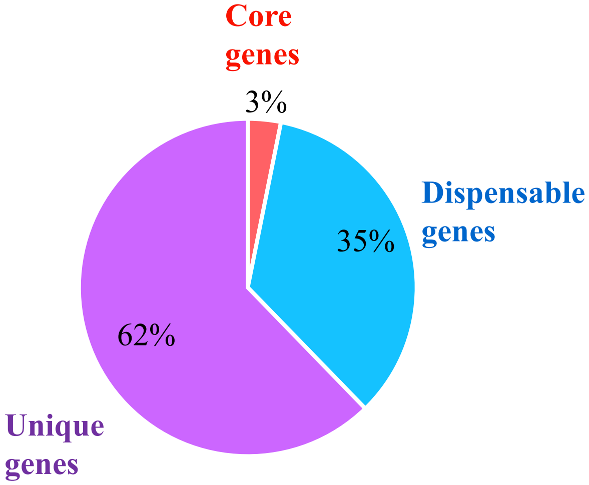

Figure S3
